# Voltage-Induced Ca^2+^ Release in Postganglionic Sympathetic Neurons in Adult Mice

**DOI:** 10.1371/journal.pone.0148962

**Published:** 2016-02-09

**Authors:** Hong-Li Sun, Wen-Chin Tsai, Bai-Yan Li, Wen Tao, Peng-Sheng Chen, Michael Rubart

**Affiliations:** 1 Riley Heart Research Center, Herman B. Wells Center for Pediatric Research, Indiana University School of Medicine, Indianapolis, Indiana, United States of America; 2 Department of Pharmacology, Harbin Medical University-Daqing, Daqing, Heilongjiang, China; 3 The Krannert Institute of Cardiology and Division of Cardiology, Department of Medicine, Indiana University School of Medicine, Indianapolis, Indiana, United States of America; 4 Division of Cardiology, Department of Medicine, Hualein Tzu-Chi General Hospital, Hualein, Taiwan; 5 Department of Biomedical Engineering, Indiana University–Purdue University at Indianapolis, Indianapolis, Indiana, United States of America; Cinvestav-IPN, MEXICO

## Abstract

Recent studies have provided evidence that depolarization in the absence of extracellular Ca^2+^ can trigger Ca^2+^ release from internal stores in a variety of neuron subtypes. Here we examine whether postganglionic sympathetic neurons are able to mobilize Ca^2+^ from intracellular stores in response to depolarization, independent of Ca^2+^ influx. We measured changes in cytosolic ΔF/F_0_ in individual fluo-4 –loaded sympathetic ganglion neurons in response to maintained K^+^ depolarization in the presence (2 mM) and absence of extracellular Ca^2+^ ([Ca^2+^]_e_). Progressive elevations in extracellular [K^+^]_e_ caused increasing membrane depolarizations that were of similar magnitude in 0 and 2 mM [Ca^2+^]_e_. Peak amplitude of ΔF/F_0_ transients in 2 mM [Ca^2+^]_e_ increased in a linear fashion as the membrane become more depolarized. Peak elevations of ΔF/F_0_ in 0 mM [Ca^2+^]_e_ were ~5–10% of those evoked at the same membrane potential in 2 mM [Ca^2+^]_e_ and exhibited an inverse U-shaped dependence on voltage. Both the rise and decay of ΔF/F_0_ transients in 0 mM [Ca^2+^]_e_ were slower than those of ΔF/F_0_ transients evoked in 2 mM [Ca^2+^]_e_. Rises in ΔF/F_0_ evoked by high [K^+^]_e_ in the absence of extracellular Ca^2+^ were blocked by thapsigargin, an inhibitor of endoplasmic reticulum Ca^2+^ ATPase, or the inositol 1,4,5-triphosphate (IP_3_) receptor antagonists 2-aminoethoxydiphenyl borate and xestospongin C, but not by extracellular Cd^2+^, the dihydropyridine antagonist nifedipine, or by ryanodine at concentrations that caused depletion of ryanodine-sensitive Ca^2+^ stores. These results support the notion that postganglionic sympathetic neurons possess the ability to release Ca^2+^ from IP_3_-sensitive internal stores in response to membrane depolarization, independent of Ca^2+^ influx.

## Introduction

Calcium ions play an important role in regulating a variety of neuronal processes, including excitability, gene transcription, synaptic plasticity, growth cone behavior, synaptogenesis, and neurotransmitter release [1,2]. Neurons use both extracellular and intracellular sources of calcium. Whereas voltage-gated calcium channels and receptor-operated channels such as the NMDA receptors enable Ca^2+^ influx from the extracellular space, inositol 1,4,5-trisphosphate (IP_3_) receptors and ryanodine receptors distributed throughout the endoplasmic reticulum membrane are responsible for releasing Ca^2+^ from its internal stores [1]. The mechanism for triggering Ca^2+^ discharge from internal stores is unknown in some cases, and it is often assumed that Ca^2+^-induced Ca^2+^ release secondary to Ca^2+^ entry is the prevailing mechanism underlying Ca^2+^ mobilization. More recent studies, however, provide evidence for the existence of a Ca^2+^ influx-independent, voltage-induced Ca^2+^ release mechanism in neurons. A skeletal muscle excitation-contraction coupling–like mechanism, wherein conformational changes of the dihydropyridine receptor directly gate the ryanodine receptor, has been reported for hippocampal neurons [3], hypothalamic magnocellular neurons [4], and ischemically injured spinal cord white matter [5]. On the other hand, voltage-induced, Ca^2+^ influx-independent, Ca^2+^ release form IP_3_-sensitive stores has been reported for insect dorsal unpaired median neurons [6]. Overall, these studies suggest the possibility that voltage-induced Ca^2+^ release from internal stores may be a more general phenomenon in neurons than previously thought.

Sympathetic ganglion neurons have been demonstrated to express both dihydropyridine-sensitive L-type calcium channels [7–10] and ryanodine receptors [11,12], suggesting the possibility that a skeletal muscle-like, voltage-induced Ca^2+^ release occurs in these cells. Here, we tested the hypothesis that sympathetic ganglion neurons in adult mice possess the ability to mobilize Ca^2+^ from internal stores in response to membrane depolarization, independent of Ca^2+^ influx. Our results provide, to the best of our knowledge, the first evidence that postganglionic sympathetic neurons are capable of releasing Ca^2+^ from internal stores in response to prolonged depolarization in the absence of extracellular calcium. Surprisingly, however, this process does not require dihydropyridine or ryanodine receptors. Rather, depolarization causes Ca^2+^ release from IP_3_-sensitive internal stores, utilizing a yet to be identified plasmalemmal voltage sensor. This process may constitute a novel mechanism coupling electrical activity to a rise in intracellular Ca^2+^ in sympathetic neurons.

## Materials and Methods

### Preparation of sympathetic neurons

Sympathetic neurons were prepared in a manner previously described [13]. DBA/J mice at 2 to 3 months of age were sacrificed by cervical dislocation. The superior and stellate ganglia were removed under stereomicroscopy and immediately placed in chilled (4–8°C) sympathetic complete medium [DME-F12 medium supplemented with sodium bicarbonate, HEPES, penicillin/streptomycin, 5% fetal bovine serum, and MITO^+^ Serum extender (Collaborative Res, Bedford, MA, USA)]. The ganglia were then incubated for 20 min at 37°C in a Earle’s Balanced Salt solution (Sigma, St. Louis, MO, USA) containing 10 U/ml papain (Worthington Biochemical Corp., Lakewood, NJ, USA), followed by a 30-min incubation in Earle’s Balanced Salt solution supplemented with 1.3 mg/ml type II collagenase (Worthington) and 2.2 mg/ml dispase II (Roche, Indianapolis, IN, USA). Cells were then dissociated by trituration with a fire-polished glass Pasteur pipette in trituration solution (sympathetic complete medium containing 1.8 mg/ml bovine serum albumin) and plated on poly-D-lysine–coated No.1 circular glass cover slips. The isolated neurons were maintained in 5% CO_2_-95% O_2_ at 37°C in trituration solution and used ~24 hours after plating. All procedures were approved by the Indiana University School of Medicine Institutional Animal Care and Use Committee.

### Cytosolic Ca^2+^ imaging

A coverslip containing sympathetic neurons was transferred to a 263-μl recording chamber (model RC-21BRFS, Warner Instruments, Hamden, CT). Cells were loaded at room temperature with fluo-4 by incubation with the acetoxymethyl (AM) ester form of the dye (fluo-4/AM; Life Technologies, Grand Island, NY) at a final concentration of 2 μM in normal Tyrode’s solution. After 20 min, cells were washed several times with dye-free Tyrode’s solution and transferred to an inverted microscope (Axioscope) equipped with a Zeiss x63 1.4 numerical aperture water immersion lens. The microscope was attached to a confocal laser-scanning unit (Zeiss LSM 510). Fluo-4 fluorescence was probed every second by illumination with 488-nm laser light and emission was detected between 500 and 550 nm. The diameter of the pinhole was set to its maximum for all measurements. Images were taken in frame-mode at a pixel density of 512 x 512. Fluorescence signals were digitized at 8-bit resolution and analyzed using Metamorph software (Molecular Probes, Sunnyvale, CA). To quantitate amplitude and time course of changes in cytosolic fluo-4 fluorescence, i.e., Ca^2+^, signal intensities of pixels located inside the neuron soma (excluding the nuclei) were measured, spatially averaged, and background corrected [F(t)]. Background fluorescence was measured as the average of a 40 x 40 pixel cell-free area outside the neuron soma of interest in each frame of every time series. Baseline fluorescence intensity (F_0_) was determined by averaging F over the 10-s interval preceding the cell’s exposure to elevated [K^+^] in the extracellular medium ([K^+^]_e_), and the time course of normalized fractional dye fluorescence [ΔF/F_0_(t)] was obtained, where ΔF equals F(t)- F_0_. Signal correction for bleaching was not necessary.

**Fig 1:**
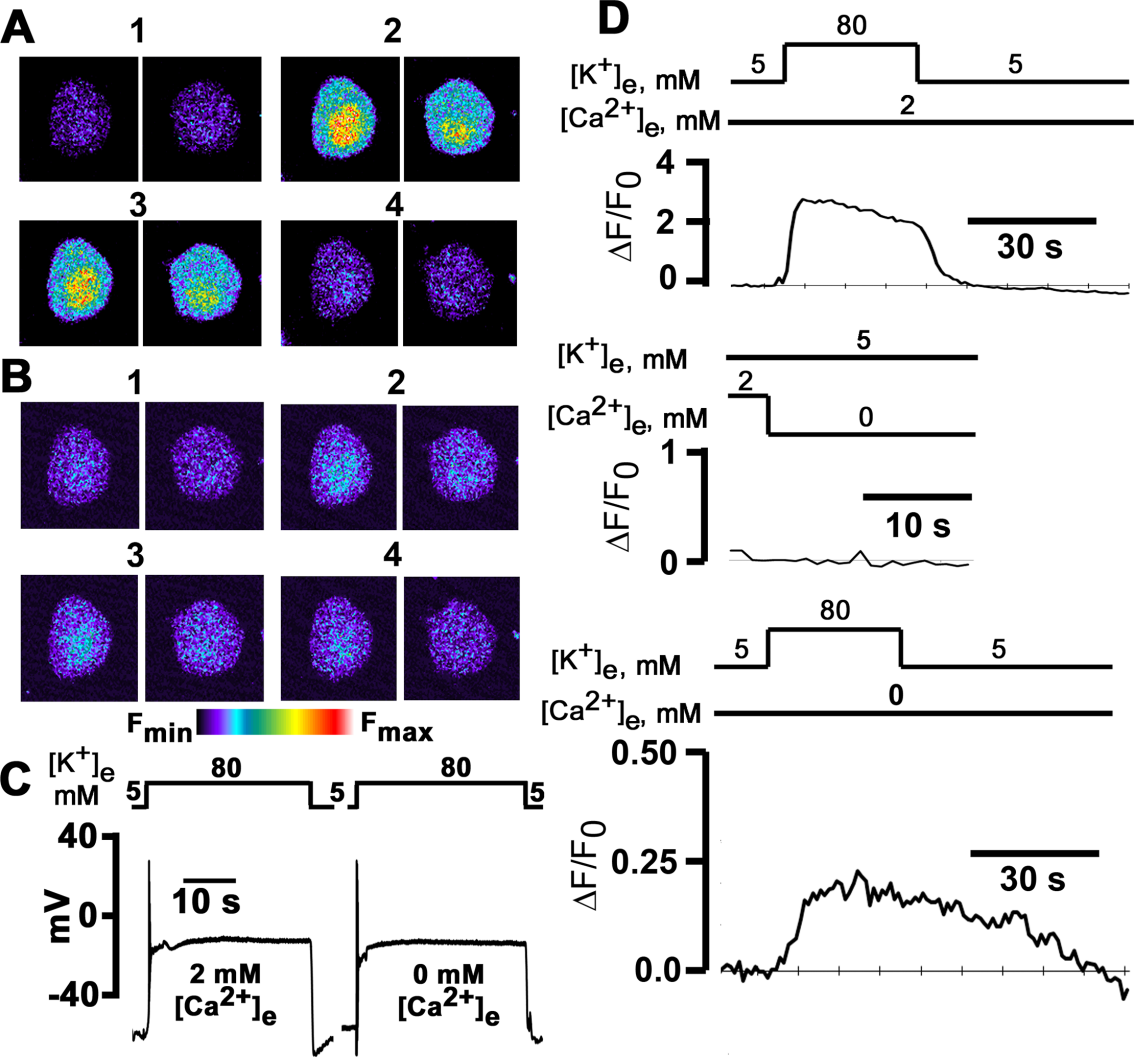
High [K^+^]_e_-induced intracellular Ca^2+^ transients in the presence and absence of extracellular Ca^2+^. **A** and **B**: *x-y* fluorescence images recorded in 24 h cultured sympathetic ganglion neurons loaded with fluo-4/AM before, during and following 30-s depolarizations with 80 mM [K^+^]_e_ in 2 mM [Ca^2+^]_e_ (**A**) and in the absence of extracellular Ca^2+^ with 200 μM EGTA added to the extracellular solution (**B**). Exposure to elevated [K^+^]_e_ evoked transient increases in both cyto- and nucleoplasmic Ca^2+^ concentrations. Images in panels 2 and 3 were acquired at 10 and 30 s after initiating the [K^+^]_e_ challenge. **C**: High [K^+^]_e_ depolarizes the membrane potential both in the presence and absence of external Ca^2+^: 80 mM [K^+^]_e_ rapidly depolarized V_m_ from a resting membrane potential of -44 mV to 1 mV in 2 mM [Ca^2+^]_e_, and from -41 mV to 0 mV in the absence of extracellular Ca^2+^. The level of depolarization was sustained for the duration of the [K^+^]_e_ challenge, both in the presence and absence of external Ca^2+^. **D**: Time courses of fractional fluorescence (ΔF/F_0_), i.e., [Ca^2+^]_i_, in the cytoplasm during exposure to 80 mM [K^+^]_e_ in the presence and absence of external Ca^2+^. ΔF/F_0_ was calculated from the fluorescence intensity measured in the whole cytoplasm for each image taken every second.

Cells were exposed to a high [K^+^] solution (40, 60, 80 or 100 mM KCl) in the presence of 2 mM Ca^2+^ for 30 s, followed by a 1-min exposure to normal Tyrode’s solution. A second 30-s exposure to the same [K^+^]_e_ in the absence of extracellular Ca^2+^ (with 200 μM EGTA added to the extracellular solution) was applied, and then the high [K^+^]_e_ solution was replaced with Ca^2+^-free normal Tyrode’s solution. The first and second applications of high [K^+^]_e_ were separated by 3-min exposures to Ca^2+^-free normal Tyrode’s solution. Bath solution exchanges were performed via manual injections (~500 μl/s) through the input port of the perfusion chamber. For each consecutive bath fluid exchange in the experimental protocol, the injected volume was 7 ml, corresponding to ~27 times the chamber volume. Solution changes were rapid, based on the fast and steady change in membrane potential achieved when cells were exposed to external solutions with elevated [K^+^] (see Fig 1C). Nominally Ca^2+^-free Tyrode’s solution contained the following (in mM): 140 NaCl, 5 KCl, 1 MgCl_2_, 0.2 EGTA, 10 HEPES, and 10 D-glucose (pH 7.4). Assuming a 50 μM total contaminating Ca^2+^, the free [Ca^2+^]_e_ was estimated to be ~ 43 nM [14,15]. For Ca^2+^-containing Tyrode’s solution, calcium was added (2 mM) and EGTA was omitted. When stimulating with elevated [K^+^]_e,_ Na^+^ was adjusted to maintain osmolarity [10,16]. Where indicated, drugs were added 20 min before and included throughout the stimulation. Pilot experiments demonstrated that the magnitude of the high [K^+^]_e_-elicited Ca^2+^ response in Ca^2+^-free bath solution gradually decreased with consecutive [K^+^]_e_ tests. Therefore, the response to one tandem only of high [K^+^]_e_ exposure (in 2 and 0 mM [Ca^2+^]_e_) was tested in an individual neuron.

### Electrophysiology

Whole-cell voltage- and current-clamp recordings were performed at room temperature using a Multiclamp 700B patch-clamp amplifier (Molecular Devices, Sunnyvale, CA, USA). Patch pipettes were pulled in a model P-97 puller (Sutter Instruments, Novato, CA, USA) from borosilicate glass capillaries and heat-polished prior to use with a Narishige MF-83 microforge (Narishige Inc., East Meadow, NY, USA). When filled with internal solution, the pipette resistance ranged from 2 to 5 MΩ. During the recording, changes in the bath solution were made by gravity driven perfusion. Pipette capacitance was zeroed on sealing. Whole-cell capacitive transients were compensated by 60–80%. Residual linear capacitive and leak currents were subtracted by the–*P*/6 method. Currents and voltages were low-pass filtered at 1 kHz and 5 kHz, respectively, using the built-in four-pole Bessel filter and sampled at 5 and 25 kHz, using a Digidata 1440A, acquired using Clampex10 and analyzed with Clampfit10 (all from Molecular Devices). Data collection was started ~2 to 3 min following membrane breakthrough.

Pipette solution for measuring whole-cell Ca^2+^ currents contained (in mM) 120 Cs-Aspartate, 1 MgCl_2_, 4 MgATP, 0.3 Na_2_GTP, 10 EGTA and 10 HEPES (pH 7.2), whereas the bath solution was composed of (in mM) 140 TEA-Cl, 2 MgCl_2_, 2 CaCl_2_, 10 glucose and 10 HEPES (pH 7.4). For measuring ion currents through voltage-gate Ca^2+^ channels in the absence of extracellular Ca^2+^, the bath solution contained (in mM) 140 NaCl, 2 MgCl_2_, 0.2 EGTA, 10 glucose and 10 HEPES (pH 7.4). For measuring action potentials, the pipette solution contained (in mM) 145 K-Aspartate, 2 MgCl_2_, 5 HEPES, 5 Na_2_ATP and 1.1 EGTA (pH 7.2), and the bath solution consisted of (in mM) 140 NaCl, 5 KCl, 1 MgCl_2_, 2 CaCl_2_, 10 HEPES and 10 glucose (pH 7.4). For Ca^2+^-free bath solutions, calcium was omitted and EGTA (0.2 mM) was added. When recording the effects of elevating [K^+^]_e_ on membrane potential, [Na^+^] was lowered to maintain osmolarity. Osmolarities of all bath and pipette solutions were adjusted to 310 and 315 mOsm, respectively, using mannitol. Membrane potentials were adjusted for liquid junction potentials unless stated otherwise.

To characterize voltage dependence of peak *I*_Ca_ and to determine the *I*_Ca._ activation curve, currents were evoked by 300-ms pulses ranging from -90 to +60 mV in steps of 5 mV. The interval between voltage steps was 10 s. The peak *I*_Ca.L_ density at each potential was plotted as a function of test voltage to generate the *I-V* curves. Activation curves were fitted with the following Boltzmann distribution equation: *G/G*_*max*_ = 1/{1+exp[*V*_*1/2*_ –*V*)/*k*]}, where *G* is the voltage-dependent calcium conductance, *G*_max_ is the maximal calcium conductance, *V*_*1/2*_ is the potential at which activation is half-maximal, *V* is the membrane potential, and *k* is the slope. *G* values were determined by the following equation: *G* = *I*_*max*_/(*V-E*_*Ca*_), where *E*_*Ca*_ is the reversal potential.

To examine steady-state inactivation, the voltage that gave maximal peak current was used for subsequent protocols. Cells were administered a series of prepulses (-90 to 40 mV) lasting 300 ms, from a holding potential of -90 mV, followed, after a 20-ms gap at -90 mV, by a 300-ms depolarization to a voltage eliciting the maximal peak current (0 mV). The interval between conditioning prepulses was 10 s. The resulting curves were normalized and fitted using the following Boltzmann distribution equation: *I/I*_*max*_ = 1/{1 + exp[(*V*–*V*_*1/2*_)/*k*]} + *C*, where *I*_*max*_ is the peak current elicited after the most hyperpolarized prepulse, *V* is the preconditioning pulse potential, and *C* is a constant.

The membrane capacitance was calculated from 5-mV hyperpolarizing and depolarizing steps (20 ms) applied from a folding potential of -70 mV according to the equation: *C*_*M*_ = τ/Δ*V* * *I*_0_/(1 –*I*_*∞*_*/I*_*0*_), where *C*_*M*_ is membrane capacitance, τ is the time constant of the capacitance current relaxation, *I*_0_ is the peak capacitive current determined by single exponential fit and extrapolation to the first sample point after the voltage step Δ*V*, and *I*_*∞*_ is the amplitude of the steady-state current during the voltage step [17]. Capacitive currents were sampled at 25 kHz and filtered at 5 kHz.

### Data analysis

Summarized data are expressed as means ± SEM. Statistical analyses were determined by paired *t*-test, unpaired *t*-test, and parametric and non-parametric one-way analyses of variance coupled with the appropriate *post hoc* analyses to determine significance (*P* < 0.05). Fisher Exact test was used to determine differences of proportions.

### Chemicals

Stock solutions of 2-aminoethoxydiphenyl borate (2-APB), nifedipine, ryanodine and thapsigargin were prepared in DMSO. The final DMSO concentration in the experimental solution did not exceed 1 μl/ml. Caffeine stock solution was prepared in ddH_2_O. Stock solution of tetrakis (2-pyridylmethyl) ethylendiamine (TPEN) was prepared in ethanol, and xestospongin C stock solution was made in PBS.

## Results

### High [K^+^]_e_-induced membrane depolarization evokes Ca^2+^ transients in mouse sympathetic ganglion neurons in the absence of extracellular Ca^2+^

Raising extracellular [K^+^] has been previously shown by others to produce stable and reproducible membrane depolarization in sympathetic ganglion neurons in culture, increasing intracellular free [Ca^2+^] in the presence of external Ca^2+^ [10]. Here, we combined [K^+^]_e_ depolarization with fluorescent Ca^2+^ imaging to examine the possibility that membrane depolarization evokes Ca^2+^ transients within isolated sympathetic ganglion neurons of adult mice not only in the presence of extracellular Ca^2+^ but also in its absence. Representative Ca^2+^ responses of two fluo-4/AM-loaded neurons elicited by consecutive 30-s exposures to 80 mM [K^+^]_e_ in the presence of 2 mM [Ca^2+^]_e_ and in Ca^2+^-free bath solution (with 200 μM EGTA added) are illustrated in Fig 1A and 1B, respectively. Elevating [K^+^]_e_ in normal Ca^2+^ gave rise to increases in global fluorescence intensity, which were larger in the nuclei than in the cytosols (Fig 1A). The fluorescence signals remained elevated throughout the exposure to high [K^+^]_e_ (panels 2 and 3 in Fig 1A) and returned to baseline values following restoration of [K^+^]_e_ (panel 4 in Fig 1A). The [K^+^]_e_ challenge was repeated in the absence of extracellular Ca^2+^ following a 3-min exposure to Ca^2+^-free normal Tyrode’s solution. As illustrated in Fig 1B, a second 30-s exposure to 80 mM [K^+^] in the absence of external Ca^2+^ gave rise to sustained elevations in intracellular Ca^2+^ (panels 2 and 3 in Fig 1B), although the magnitude of the effect was markedly reduced compared to the 2 mM [Ca^2+^]_e_ condition. Fluorescence signal intensity recovered upon restoration of [K^+^]_e_ in the continued absence of extracellular Ca^2+^.

To make certain that membrane depolarization evoked by 80 mM [K^+^]_e_ was maintained at similar potentials in the presence and absence of external Ca^2+^, the membrane potential was recorded from isolated sympathetic neurons before and during the 30-s depolarization. A representative example is shown in Fig 1C. Both in the presence and absence of extracellular Ca^2+^, high [K^+^]_e_ rapidly depolarized the neuron following the solution change, and remained stable while [K^+^]_e_ = 80 mM. The magnitude of depolarization appeared similar under both conditions. After [K^+^]_e_ was restored to 5 mM, membrane potential exhibited a transient afterhyperpolarization in 2 mM [Ca^2+^]_e_. On average, the degree of membrane depolarization evoked by 80 mM [K^+^]_e_ in 0 mM [Ca^2+^]_e_ was slightly less than that in 2 mM [Ca^2+^]_e_ (see Table 1).

**Table 1:** Membrane potentials of sympathetic ganglion neurons at rest and during depolarization with elevated [K^+^]_e_ in normal [Ca^2+^]_e_ and in the absence of extracellular Ca^2+^ (with 200 μM EGTA added to the bath solution). Values are expressed as means ± SEM.

[K^+^]_e_, mM	2 mM [Ca^2+^]_e_, mV	0 mM [Ca^2+^]_e_, mV
5	-61.8 ± 4.4	-59.9 ± 3.1
40	-31.8 ± 1.4	-31.2 ± 0.7
60	-21.9 ± 0.9	-23.2 ± 0.9
80	-15.7 ± 0.5	-18.5 ± 0.8*
100	-11.5 ± 0.6	-14.2 ± 0.8*

*P<0.05 versus 2 mM [Ca^2+^]_e_ by paired *t*-test or Wilcoxon Signed Rank test.

**Fig 2:**
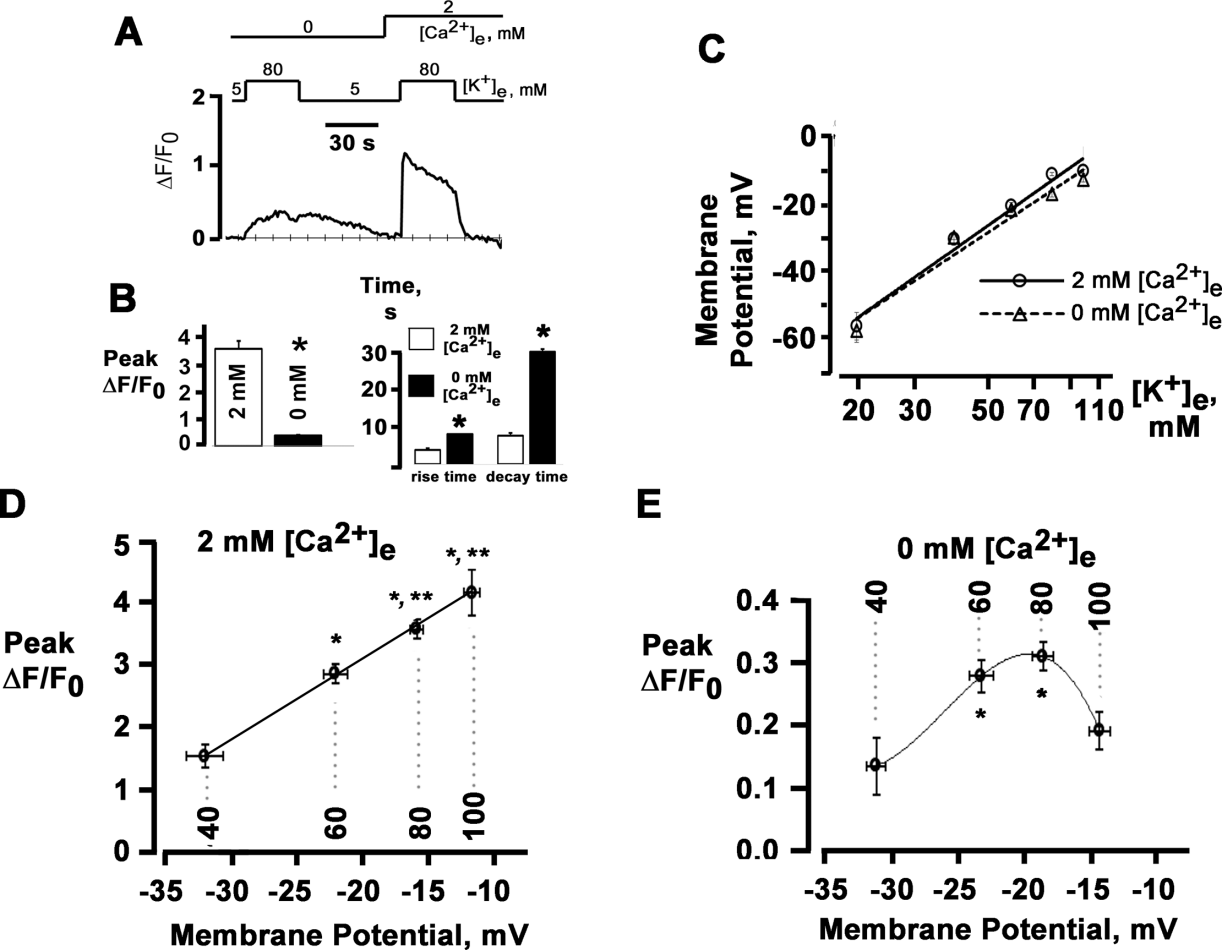
Sympathetic neurons possess the ability to release Ca^2+^ from intracellular stores in response to prolonged membrane depolarization in the absence of extracellular Ca^2+^. **A**: Time course of cytosolic fluo-4 ΔF/F_0_ in a sympathetic neuron in response to a 30-s exposure to 80 mM [K^+^]_e_ in 2 mM [Ca^2+^]_e_ and a 30-s exposure to 80 mM [K^+^]_e_ in Ca^2+^-free solution, followed by a second 30-s application of 80 mM [K^+^]_e_ in 2 mM [Ca^2+^]_e_ applied 70 seconds after the Ca^2+^ transient was elicited in Ca^2+^- free solution. **B**: Bar graphs of peak magnitude of the cytosolic ΔF/F_0_ transients (left panel) and their rise and decay times during exposure to 80 mM [K^+^]_e_ in the presence and absence of extracellular Ca^2+^ (with 200 μM EGTA added). Rise times and decay times were measured as the intervals from 10% to 90% and from 90% to 10%, respectively, of peak ΔF/F_0_. Values are mean ± SEM from 48 to 87 cells. *P < 0.0001 versus 2 mM [Ca^2+^]_e_, paired *t*-test. **C**: Plots of sympathetic neuron membrane potential as a function of [K^+^]_e_. Values are mean ± SEM (n = 5–8 experiments for each [K^+^]_e_ studied). Lines are linear fits with slopes of 67.5 mV (2 mM [Ca^2+^]_e_) and 62.2 mV (0 mM [Ca^2+^]_e_) per 10-fold change in [K^+^]_e_. **D** and **E**: Peak magnitude of cytosolic ΔF/F_0_ transients as a function of membrane potential. Shown are the relationships between membrane potential and peak ΔF/F_0_ amplitude in response to 30-s exposures to 40, 60, 80 or 100 mM [K^+^]_e_ in 2 mM [Ca^2+^]_e_ (**D**) or in the absence of extracellular Ca^2+^ (**E**). Values are mean ± SEM (n = 18–87 cells for each [K^+^]_e_). Solid lines represent best fits of the data to a linear function (D) and a polynomial function (E). Dashed lines denote membrane potentials generated by each [K^+^]_e_. **B**: * *P* < 0.001 versus 40 mM [K^+^]_e_; ** *P* < 0.03 versus 60 mM [K^+^]_e_. **C**: * *P* < 0.05 versus 40 mM [K^+^]_e_; repeated measures ANOVA and Dunn’s method for multiple comparisons.

Next, we sought to quantitate the high [K^+^]_e_-evoked changes in cytosolic Ca^2+^ level, using ΔF/F_0_ as a measure. Plots of ΔF/F_0_ as a function of time for one of the neurons in Fig 1A and 1B are displayed in Fig 1D. The neuron responds to high [K^+^]_e_ exposure with an initial peak followed by a slow, spontaneous decay, which was seen in all cells tested, despite the fact that the depolarization of the plasma membrane was maintained throughout the 30-s [K^+^]_e_ challenge. Increases in ΔF/F_0_ typically resolved within less than 15 s after restoration of [K^+^]_e_. Replacement of normal Tyrode’s solution with Ca^2+^-free solution did not evoke significant changes in cytosolic [Ca^2+^] (Fig 1D, middle panel), indicating that the solution change *per se* does not contribute to the rise in fluo-4 fluorescence seen in response to elevated [K^+^]_e_ in the absence of external Ca^2+^. A 30-s exposure of the same neuron to 80 mM [K^+^]_e_ in Ca^2+^-free bath solution evoked a low-amplitude Ca^2+^ transient which exhibited markedly slower rise and decay kinetics compared to the transient in 2 mM [Ca^2+^]_e_ (Fig 1D, lower panel). Overall, 47 (78%) of a total of 60 neurons responded with a rise in cytosolic [Ca^2+^] in the absence of extracellular Ca^2+^. To assess whether loss of cellular viability gives rise to these marked differences in Ca^2+^ transient properties, a subset of neurons were re-exposed to high [K^+^]_e_ in normal [Ca^2+^]_e_ following the [K^+^]_e_ test in Ca^2+^ free conditions. A representative ΔF/F_0_ (t) tracing is shown in Fig 2A. After raising [Ca^2+^]_e_ to 2 mM, the peak amplitude of the high [K^+^]_e_-evoked Ca^2+^ transient clearly exceeded that of the preceding transient recorded in the absence of external Ca^2+^, and its time course was indistinguishable from that of a response typically seen during initial exposures to high [K^+^]_e_ in 2 mM [Ca^2+^]_e_. Identical observations were made in 7 cells. These results prove that the neurons remain viable and support the notion that the differences in Ca^2+^ transient properties between 2 and 0 mM [Ca^2+^]_e_ are unlikely to result from unspecific effects of the exposure to the Ca^2+^ free bath solution. At 80 mM [K^+^]_e_, the average peak Ca^2+^ transient amplitude was markedly smaller in the absence than in the presence of external Ca^2+^ and their average rise and decay times were markedly prolonged compared to Ca^2+^ transients evoked in normal [Ca^2+^]_e_ (Fig 2B). Overall, these results suggest that a subpopulation of sympathetic neurons possess the ability to release Ca^2+^ from intracellular stores in response to prolonged membrane depolarization in the absence of extracellular Ca^2+^.

To relate the magnitude of the Ca^2+^ responses to membrane depolarization, we next recorded Ca^2+^ transients during exposure to various K^+^ concentrations. In a parallel series of experiments, we measured membrane voltage in isolated neurons, using external solutions identical to those in the Ca^2+^ imaging experiments.

Exposure to 40, 60, 80 and 100 mM [K^+^]_e_ in 2 mM [Ca^2+^]_e_ caused progressively increasing membrane depolarizations (Table 1), yielding a slope of 67.5 mV per 10-fold change in [K^+^]_e_ (solid line in Fig 2C). Plotting the magnitude of peak ΔF/F_0_ transient amplitude against membrane potential (Fig 2D) revealed a linear increase over a range between -31.8 and -11.5 mV. Removal of Ca^2+^ from the extracellular solution did not significantly alter the resting membrane potential in 5 mM [K^+^] Tyrode’s solution (Table 1). Progressive elevations in [K^+^]_e_ caused depolarizations that were of similar magnitude to those seen in 2 mM [Ca^2+^]_e_ (Table 1), giving rise to a slope of 62.2 mV per 10-fold change in [K^+^]_e_ (dashed line in Fig 2C). Over the voltage range examined (-31.2 to -14.2 mV), the magnitude of peak ΔF/F_0_ transient amplitude displayed an inverse U-shaped dependence on membrane potential as shown in Fig 2E, which is in sharp contrast to the linear rise in peak ΔF/F_0_ amplitude that was observed in response to progressively increasing membrane depolarizations in 2 mM [Ca^2+^]_e_ (Fig 2D).

**Fig 3:**
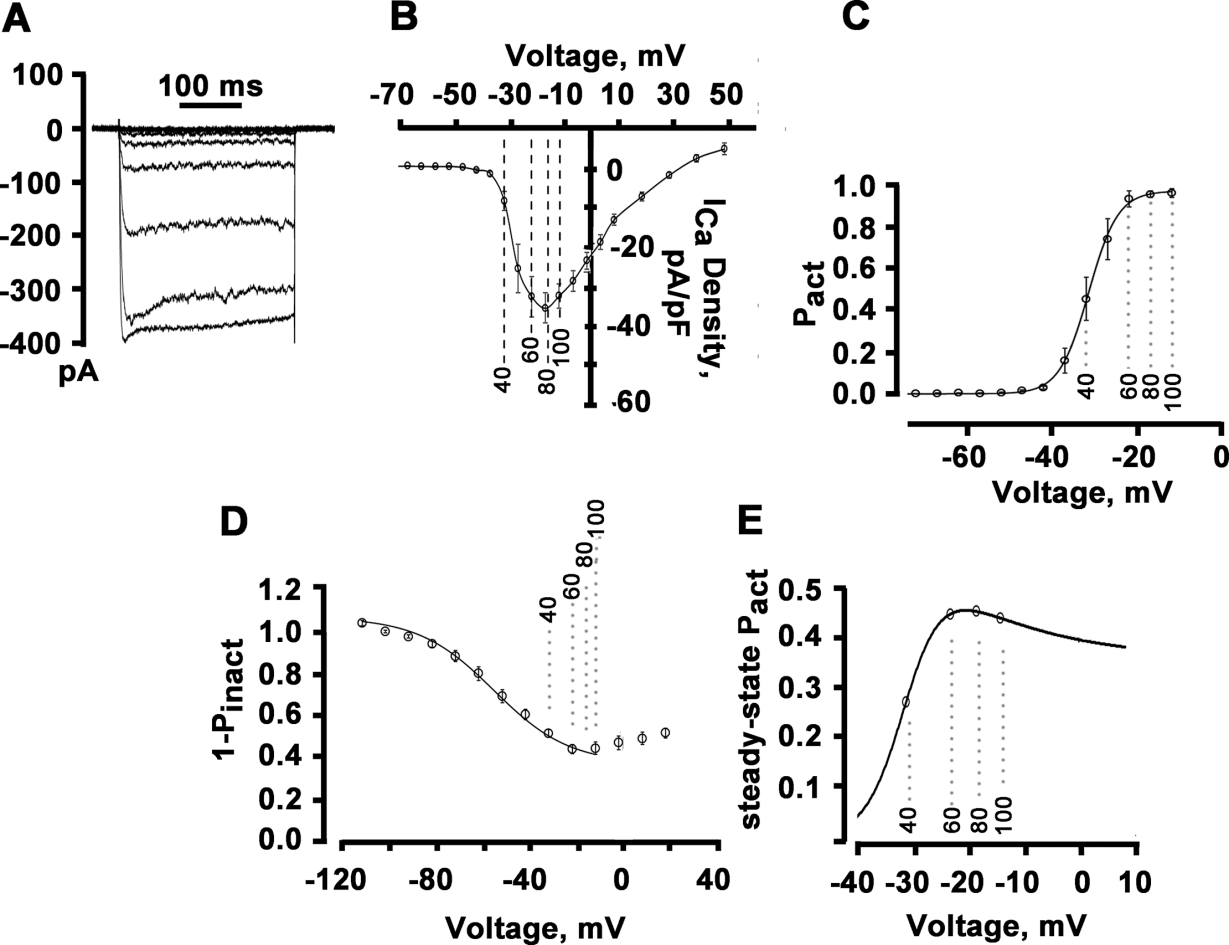
Peak magnitude of cytosolic ΔF/F_0_ transients as a function of calcium channel activity. **A**: Representative traces of whole-cell *I*_Ca_ recordings with 2 mM [Ca^2+^]_e_. Currents were elicited using 300-ms depolarizations ranging between -50 and +10 mV in 5-mV steps. Pulse interval was 10 s. **B**: Peak *I*_Ca_−voltage relationship. Current amplitudes were normalized to cell capacitance and plotted as mean values. Error bars represent SEM (n = 11 cells). Dashed lines indicate voltages generated by each [K^+^]_e_ in 2-mM [Ca^2+^]_e_ bath solutions. **C**: Voltage-dependence of steady-state *I*_Ca_ activation, *P*_act_. Values are mean ± SEM for 11 cells. The solid line represents the mean of the best fit to each cell by a Boltzmann distribution, with *V*_*1/2*_ and *k* values of -31.2 mV and 3.4 mV, respectively. **D**: Voltage-dependence of steady-state *I*_Ca_ inactivation, (1 –*P*_inact_). For measuring voltage-dependence of inactivation, a paired-pulse voltage protocol was used consisting of a 300-ms conditioning prepulse to voltages from -90 to 40 mV followed, after a 20-ms gap at -90 mV, by a 300-ms test pulse to 0 mV. Holding potential was -90 mV and the interval between conditioning prepulses as 10 s. For generating inactivation curve, the peak amplitudes of currents evoked by the test pulse were normalized to the current evoked during each prepulse and plotted as a function of prepulse potential. Solid line is the mean of the best fit of the descending portion of the inactivation-voltage relationship (i.e., between -111.9 and 18.1 mV) to a Boltzmann function, with *V*_*1/2*_ and *k* values of 55.3 mV and 16.3 mV, respectively. Values are mean ± SEM (n = 8 cells). **E**: Calculated voltage-dependence of steady-state *P*_act_ of high voltage-gated Ca^2+^ channels in sympathetic ganglion neurons. The curve shows the theoretical steady-state *P*_act_ at any potential, using the Boltzmann values for the amount of available current and the amount of current inactivation. The maximum available current was set to 1. Circles denote values for steady-state *P*_act_ at voltages generated by each [K^+^]_e_ in Ca^2+^-free bath solutions.

The increase in the magnitude of Ca^2+^ transient amplitude with increasing depolarization in the presence of 2 mM extracellular Ca^2+^ suggested a role of voltage-gated Ca^2+^ channel activity in governing this relationship, as had been described previously in mammalian postganglionic sympathetic neurons [10,18]. Accordingly, to relate Ca^2+^ transient properties to Ca^2+^ channel activity, we measured whole-cell Ca^2+^ currents (*I*_Ca_) over a wide range of voltages including those generated by the lowest and highest [K^+^]_e_ used in the Ca^2+^ imaging experiments. Short depolarizations evoked Ca^2+^ currents exhibiting no, or very little, inactivation (Fig 3A). A plot of peak *I*_*Ca*_ as a function of voltage is shown in Fig 3B, exhibiting the typical bell-shaped dependence on membrane potential. The descending portion of the *I*_Ca_-*V* curve largely coincided with the voltage range over which peak ΔF/F_0_ increased (see Fig 2D), suggesting that the membrane potential dependence of peak *I*_Ca_ controls Ca^2+^ transient magnitude over the range of membrane depolarizations studied here. This observation is in agreement with previous studies [10,18]. To examine whether depolarization-induced changes in Ca^2+^ channel gating underlie the membrane potential-dependence of peak ΔF/F_0_ in the absence of external Ca^2+^, we next determined the voltage-dependence of Ca^2+^ channel activation. The result is shown in Fig 3C. The probability of the channel of being activated (*P*_act_) increased in a sigmoidal fashion as the membrane potential became more depolarized. This behavior contrasts with the inverse U-shaped dependence of peak ΔF/F_0_ on voltage in Ca^2+^-free bath solution (Fig 2E), suggesting that membrane potential-dependent changes in peak *P*_act_ of voltage-activated Ca^2+^ channels do not constitute the trigger for Ca^2+^ release in 0 mM [Ca^2+^]_e_.

The slow kinetics of increase in [Ca^2+^]_i_ during sustained membrane depolarization in the absence of external Ca^2+^ (see Fig 2B) suggested the possibility that Ca^2+^ release is regulated by the steady-state activity of voltage-gated Ca^2+^ channels. The steady-state probability of a channel of being activated is the product of peak *P*_act_ (obtained from the activation curve in Fig 3C) and the probability of not being inactivated (1-*P*_inact_), i. e., steady-state *P*_act_ = (peak *P*_act_) * (1 –*P*_inact_). Accordingly, we next determined the voltage-dependence of *I*_Ca_ inactivation. Channel inactivation displayed a U-shaped dependence on voltage (Fig 3D). Fits of the descending portion of the inactivation curve to a Boltzmann distribution revealed *V*_*1/2*_ and *k* values of -55.3 mV and 16.3 mV, respectively. Fig 3E shows the relationship between membrane potential and steady-state *P*_act_. Negative to -20 mV, steady-state *P*_act_ increased steeply, whereas it decreased slightly at less negative potentials. Thus, steady-state activation of voltage-gated Ca^2+^ channels and peak ΔF/F_0_ in the absence of external Ca^2+^ shared a similar, i.e., inverse U-shaped, dependence on membrane potential over identical voltage ranges, suggesting that magnitudes of changes in the depolarization-induced [Ca^2+^]_i_ in 0 mM [Ca^2+^]_e_ are controlled by the steady-state gating of voltage-activated Ca^2+^ channels.

### Depolarization in Ca^2+^ free solution induces Ca^2+^ release from IP_3_-, but not ryanodine-, sensitive internal stores

**Fig 4:**
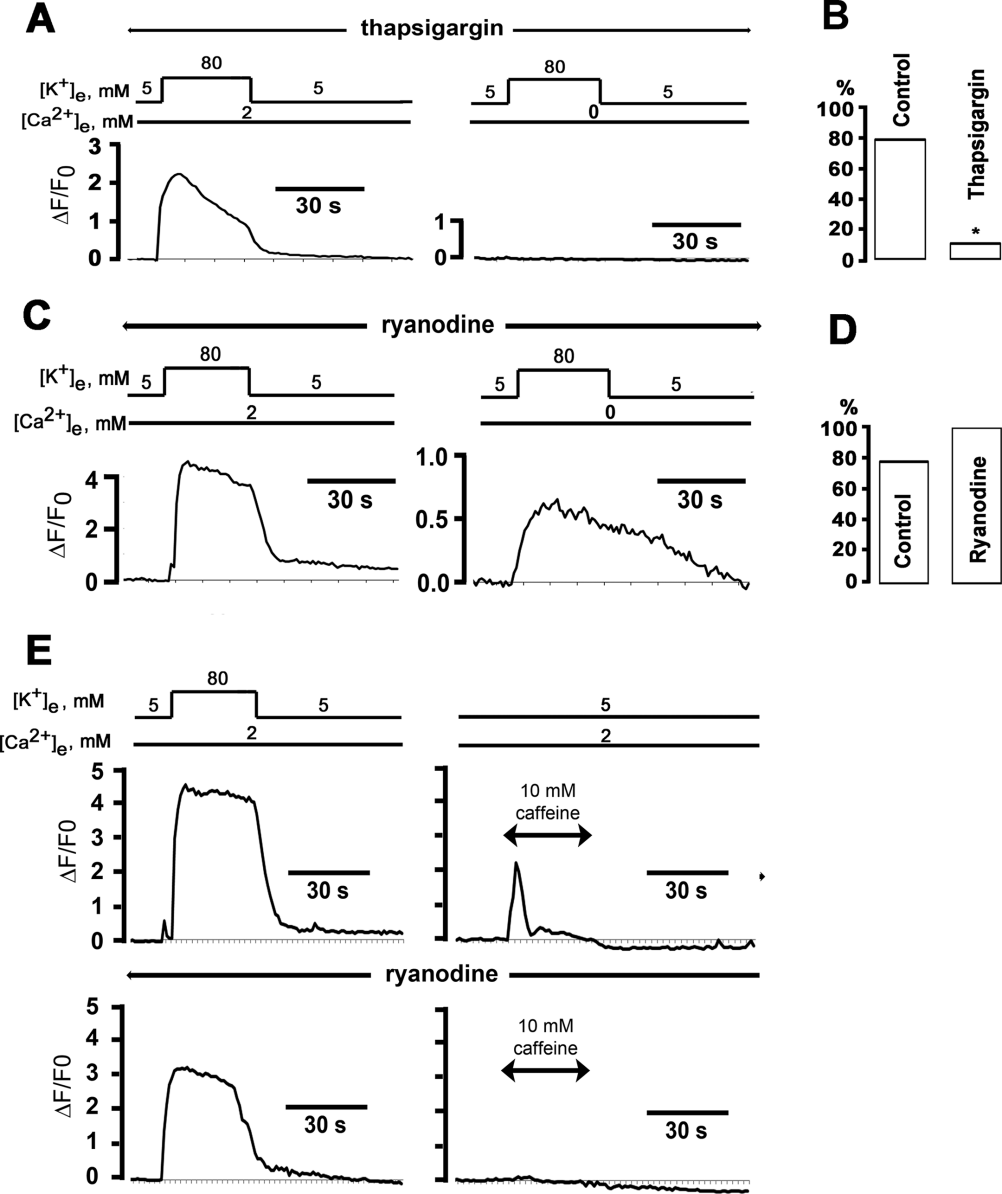
Thapsigargin, but not ryanodine, suppresses high [K^+^]_e_-induced cytosolic Ca^2+^ transients in the absence, but not in the presence, of extracellular Ca^2+^. **A**: Time course of cytosolic ΔF/F_0_ during 30-s exposures to 80 mM [K^+^]_e_ in 2 mM [Ca^2+^]_e_ (left panel) and in the absence of external Ca^2+^. To deplete internal Ca^2+^ stores, cells were continuously incubated with thapsigargin (1 μM) starting 20 min before the first exposure to elevated [K^+^]_e_. **B**: Percentage of cells exhibiting [Ca^2+^]_i_ transients both in 2 mM and 0 mM [Ca^2+^]_e_; * *P* < 0.001 versus control by Fisher Exact test (60 cells for control and 19 cells for thapsigargin). **C**: Time course of changes in cytosolic ΔF/F_0_ elicited by consecutive 30-s exposures to 80 mM [K^+^]_e_ in normal [Ca^2+^]_e_ (upper panel) and in Ca^2+^-free bath solution (with 200 μM EGTA added). The cell was continuously incubated with ryanodine (20 μM) starting 20 min before the first [K^+^]_e_ challenge. **D**: Percentage of cells exhibiting [Ca^2+^]_i_ transients both in 2 mM and 0 mM [Ca^2+^]_e_; *P* = non-significant versus control by Fisher’s Exact test (60 cells for control and 14 cells for ryanodine). **E**: Ryanodine depletes caffeine-sensitive internal Ca^2+^ stores in postganglionic sympathetic neurons. Time course of changes in cytosolic ΔF/F_0_ in response to a 30-s exposure to 80 mM [K^+^]_e_ followed by a 30-s exposure to caffeine (upper panel). Preincubation with 20 μM ryanodine abrogated the caffeine-, but not the high [K^+^]_e_-, induced cytosolic Ca^2+^ transient (lower panel). Caffeine responses in the presence and absence of ryanodine were monitored in two different cells.

To investigate the role of Ca^2+^ release from internal stores in mediating depolarization-induced increases in cytosolic [Ca^2+^], we next assessed the effects of the Ca^2+^-ATPase inhibitor thapsigargin (1 μM) on high [K^+^]_e_-evoked Ca^2+^ responses in isolated sympathetic neurons. Neurons were pre-incubated with thapsigargin for 20 min while being loaded with fluo-4/AM and then subjected to Ca^2+^ imaging in the continued presence of the inhibitor. Representative plots of ΔF/F_0_ as a function of time are shown in Fig 4. A 30-s exposure to 80 mM [K^+^]_e_ in the presence of extracellular Ca^2+^ caused an increase in cytosolic [Ca^2+^]. A second 30-s exposure to 80 mM [K^+^] in the absence of external Ca^2+^ did not evoke a significant increase in intracellular [Ca^2+^]. Overall, only 2 (10.5%) of 19 thapsigargin-treated neurons developed Ca^2+^ transients during exposure to elevated [K^+^]_e_ in the absence of extracellular Ca^2+^ compared to 47 (78%) of 60 non-treated cells (*P* < 0.001; Fig 4B). To make certain that membrane depolarization evoked by 80 mM [K^+^]_e_ was maintained at similar potentials among thapsigargin-treated and non-treated neurons in the study, we compared membrane potentials recorded from isolated sympathetic neurons before and during high [K^+^]_e_-induced depolarizations in the presence of the drug with those recorded in its absence. We found no significant differences in resting membrane potential or potentials obtained during the 80-mM [K^+^]_e_ challenges either in normal Ca^2+^ or Ca^2+^-free bath solutions (*P* > 0.05) among the two experimental groups (Table 2). Collectively, these results indicate that thapsigargin-induced abolition of high [K^+^]_e_-Ca^2+^ transients in 0 mM [Ca^2+^]_e_ does not result from a collapse of the membrane potential but rather supports the notion that depolarization elicits Ca^2+^ release from intracellular stores independently of Ca^2+^ influx.

**Table 2:** Membrane potentials of postganglionic sympathetic neurons at rest and during exposure to 80 mM [K^+^]_e_ in normal [Ca^2+^]_e_ and in the absence of extracellular Ca^2+^ (with 200 μM EGTA added to the bath solution). Values are expressed as means ± SEM. n indicates the number of cells. There were no statistically significant differences in the membrane potential between the treatment groups (*P* > 0.05 by One Way Analysis of Variance).

Treatment	Control (mV, n = 6)	1 μM Thapsigargin (mV, n = 9)	20 μM 2-APB (mV, n = 4)
5 mM [K^+^]_e_ + 2 mM [Ca^2+^]_e_	-61.8 ± 4.4	-62.1 ± 2.6	-67.7 ± 3.1
80 mM [K^+^]_e_ + 2 mM [Ca^2+^]_e_	-15.7 ± 0.5	-15.3 ± 1.4	-11.1 ± 2.2
5 mM [K^+^]_e_ + 0 mM [Ca^2+^]_e_	-59.9 ± 3.1	-55.6 ± 2.2	-63.4± 4.0
80 mM [K^+^]_e_ + 0 mM [Ca^2+^]_e_	-18.5 ± 0.8	-18.8 ± 1.3	-14.6 ± 2.0

Depolarization-evoked cytosolic Ca^2+^ transients in the absence of extracellular Ca^2+^ could result from conformational coupling between L-type Ca^2+^ channels and ryanodine receptors, as in skeletal muscle excitation-contraction coupling [4,19]. Accordingly, we examined the effect of pharmacological blocking Ca^2+^ release from ryanodine-sensitive stores on depolarization-induced Ca^2+^ release. We found that pretreating neurons for 20 min with ryanodine (20 μM) spared the high [K^+^]_e_-induced Ca^2+^ responses both in the presence and absence of extracellular Ca^2+^ (Fig 4C). Overall, there was no significant difference in the proportion of neurons exhibiting [K^+^]-induced Ca^2+^ elevations in 0 mM [Ca^2+^]_e_ between the ryanodine [14 (100%) of 14 cells] and the control group [47 (78%) of 60 cells; *P* > 0.05; Fig 4D]. As a probe for ryanodine receptor function, we applied caffeine (10 mM), an agonist of ryanodine receptor-mediated Ca^2+^ release, and monitored intracellular [Ca^2+^]. Exposures to 80 mM [K^+^]_e_ and caffeine both reversibly elevated cytosolic [Ca^2+^]. Pretreatment with 20 μM ryanodine abrogated the caffeine response, but did not affect the high [K^+^]_e_-induced response, which is consistent with previous studies (Fig 4E) [10], but differs from other studies demonstrating changes of [Ca^2+^]_i_ transient amplitude and/or kinetics by ryanodine [14,18]. Identical observations were made in 3 more cells. Thus, depolarization-evoked Ca^2+^ release in sympathetic ganglion neurons does not require ryanodine receptors, ruling out a skeletal muscle excitation-contraction coupling-like mechanism.

**Fig 5:**
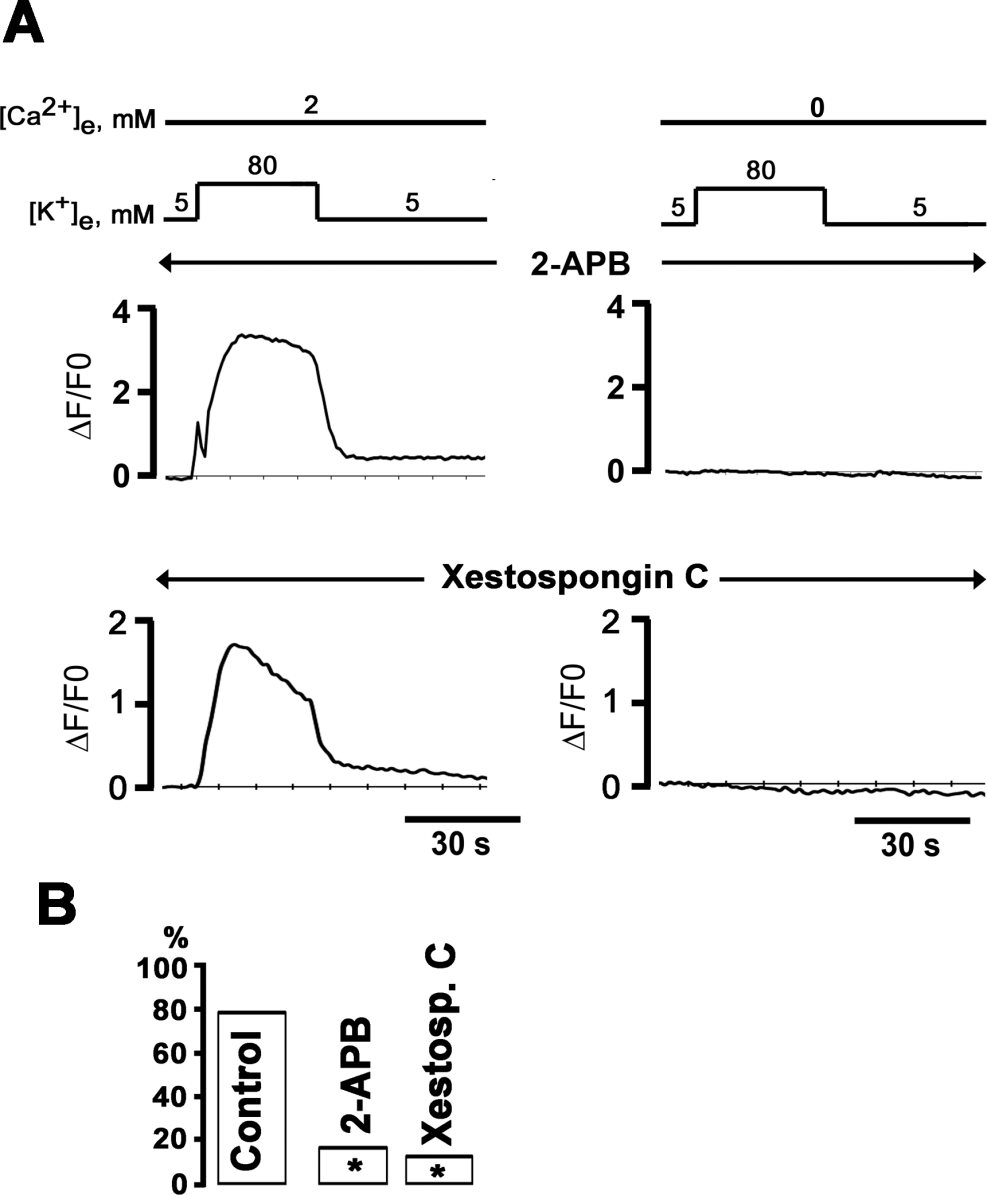
Pharmacological inhibitors of IP_3_ receptors abrogate high [K^+^]_e_-induced Ca^2+^ transients in Ca^2+^-free bath solution. **A**: Representative time courses of changes in cytosolic ΔF/F_0_ in response to two consecutive 30-s exposures to 80 mM [K^+^]_e_ in 2 mM [Ca^2+^]_e_ (left panels) and in Ca^2+^-free bath solution (with 200 μM EGTA added; right panels) following 20-min incubation with 20 μM 2-APB or 10 μM xestospongin C. **B**: Percentage of neurons exhibiting [Ca^2+^]_i_ transients both in 2 mM and 0 mM [Ca^2+^]_e_; * *P* < 0.001 versus control by Fisher’s Exact test (60, 6 and 16 cells for control, 2-APB and xestospongin C, respectively).

We then examined the contribution of Ca^2+^ release from IP_3_-sensitive Ca^2+^ stores to depolarization-induced Ca^2+^ transients in the absence of external Ca^2+^. Previous studies had demonstrated that depolarization of insect dorsal unpaired neurons in the absence of external Ca^2+^ triggers Ca^2+^ release from IP_3_-sensitive stores [6]. To determine whether IP_3_ receptors contribute to depolarization-evoked Ca^2+^ signaling in sympathetic ganglion neurons, we pharmacologically blocked release from IP_3_-sensitive stores and then measured 80 mM [K^+^]_e_-induced changes in cytosolic [Ca^2+^]. We found that pretreating the neurons for 20 min with the IP_3_ receptor inhibitors 2-aminoethoxydiphenyl borate (2-APB; 20 μM) or xestospongin C (10 μM) abolished the [K^+^]_e_-evoked Ca^2+^ transient in the absence, but not in the presence, of extracellular Ca^2+^ (Fig 5A). Overall, exposure to elevated [K^+^]_e_ in 0 mM [Ca^2+^]_e_ induced Ca^2+^ transients in only 1 (17%) of 6 2-APB–treated and 2 (10.5%) of 16 xestospongin C- treated neurons, compared to 47 (78%) of 60 control neurons (*P* = 0.001; Fig 5B). The prevalence of non-responding cells was similar following treatment with 2-APB or xestospongin C (*P* > 0.05). Further, we found no significant differences in resting membrane potential or potentials obtained during the 80-mM [K^+^]_e_ challenges either in normal [Ca^2+^]_e_ or Ca^2+^-free bath solutions (*P* > 0.05) between control neurons and neurons following IP_3_ receptor blockade (Table 2). Collectively, these results support the notion that membrane depolarization in the absence of extracellular Ca^2+^ triggers Ca^2+^ release from IP_3_-sensitive internal stores, leading to slow rises in cytosolic [Ca^2+^].

### Depolarization-evoked Ca^2+^ release in the absence of extracellular Ca^2+^ does not require L-type Ca^2+^ channels

**Fig 6:**
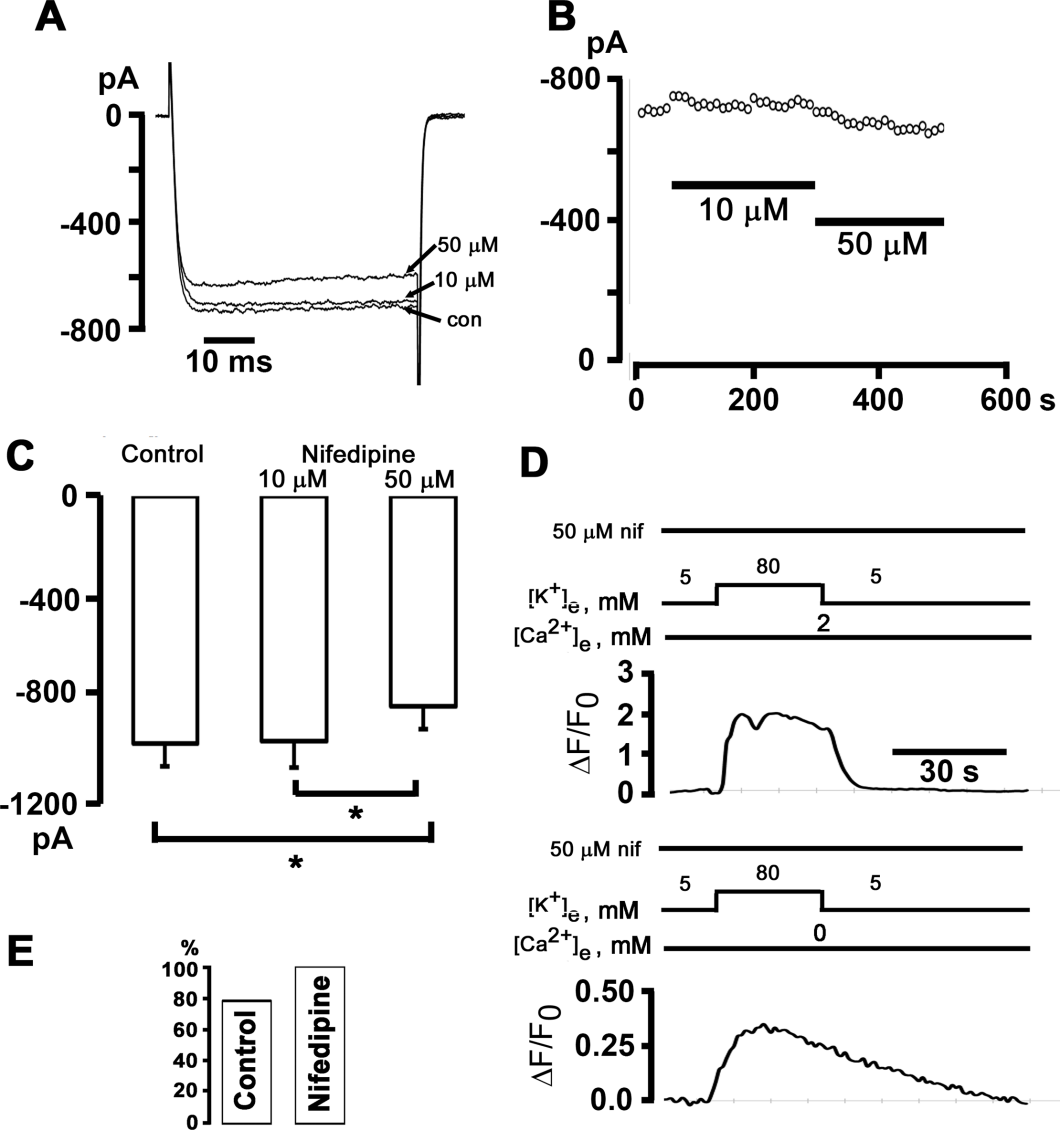
The 1,4-dihydropyridine antagonist of L-type Ca^2+^ channels, nifedipine, does not affect Ca^2+^ transients elicited by exposure to elevated [K^+^]_e_. **A** and **B**: The response of peak *I*_*Ba*_ in a postganglionic sympathetic neuron to application of 10 and 50 μM nifedipine. *I*_*Ba*_ was activated by depolarizations from -80 mV to 10 mV every 10 seconds. Representative current traces are shown in A. **C**: Bar graphs represent the average peak *I*_*Ba*_ in control and in 10 and 50 μM nifedipine. Values are from 10 cells. *P < 0.05, RM ANOVA on ranks followed by Student-Newman-Keuls Method for multiple comparisons. **D**: Exemplar time courses of changes in cytosolic ΔF/F_0_ elicited by 80 mM [K^+^]_e_ in the presence (upper panel) and absence of external Ca^2+^. The cell was continuously bathed in 50 μM nifedipine starting 20 min before the first [K^+^]_e_ test. **E**: Percentage of cells exhibiting [Ca^2+^]_i_ transients both in 2 mM and 0 mM [Ca^2+^]_e_; *P* = non-significant versus control by Fisher Exact test (60 cells for control and 10 cells for nifedipine).

Voltage-dependent changes in L-type Ca^2+^ channel (dihydropyridine receptor) gating have previously been identified as the mechanism linking membrane depolarization to Ca^2+^ release from IP_3_-sensitive stores in skeletal myotubes in the absence of extracellular Ca^2+^ [20]. If an identical mechanism is at work in sympathetic ganglion neurons, we would expect the 1,4-dihydropyridine L-type Ca^2+^ channel antagonist nifedipine, which acts on the channel by immobilizing its gating charge, to affect depolarization-evoked Ca^2+^ transients. Measurements of whole-cell currents through high voltage-activated Ca^2+^ channels (with Ba^2+^ as charge carrier) revealed that a high concentration of nifedipine (50 μM) was required to achieve a small (~-20%), yet significant, reduction in current amplitude (Fig 6A–6C). The finding that nifedipine at 10 μM did not block *I*_Ba_ strongly suggests a lack of functional L-type Ca^2+^ channels in adult sympathetic ganglion neurons, whereas the significant reduction of *I*_Ba_ at 50 μM nifedipine may reflect inhibition of Ca_v_2.x channels [21]. Alternatively, the insensitivity of whole-cell *I*_Ba_ to inhibition by nifedipine may suggest that adult sympathetic ganglion neurons express the skeletal muscle isoform of the L-type Ca^2+^ channel which has been shown previously to require nearly 50 μM nifedipine for complete block [22]. We found that pretreating neurons for 20 min with a high concentration of nifedipine (50 μM) spared the high [K^+^]_e_-induced Ca^2+^ responses both in the presence and absence of extracellular Ca^2+^ (Fig 6D). The lack of a significant effect of nifedipine on the magnitude of depolarization-evoked Ca^2+^ transients in the presence of normal [Ca^2+^]_e_ is consistent with the notion that Ca^2+^ entry via L-type Ca^2+^ channels does not contribute noticeably to the rise in global [Ca^2+^] in our study. Overall, there was no significant difference in the proportion of neurons exhibiting [K^+^]-induced Ca^2+^ elevations in 0 mM [Ca^2+^]_e_ between the nifedipine [10 (100%) of 10 cells] and the control group [47 (78%) of 60 cells; *P* > 0.05; Fig 6E]. Collectively, these results support the notion that high [K^+^]_e_-evoked Ca^2+^ release in the absence of extracellular Ca^2+^ does not require L-type Ca^2+^ channels.

### Depolarization-induced [Ca^2+^]_i_ transients in the absence of extracellular calcium are not blocked by extracellular cadmium

**Fig 7:**
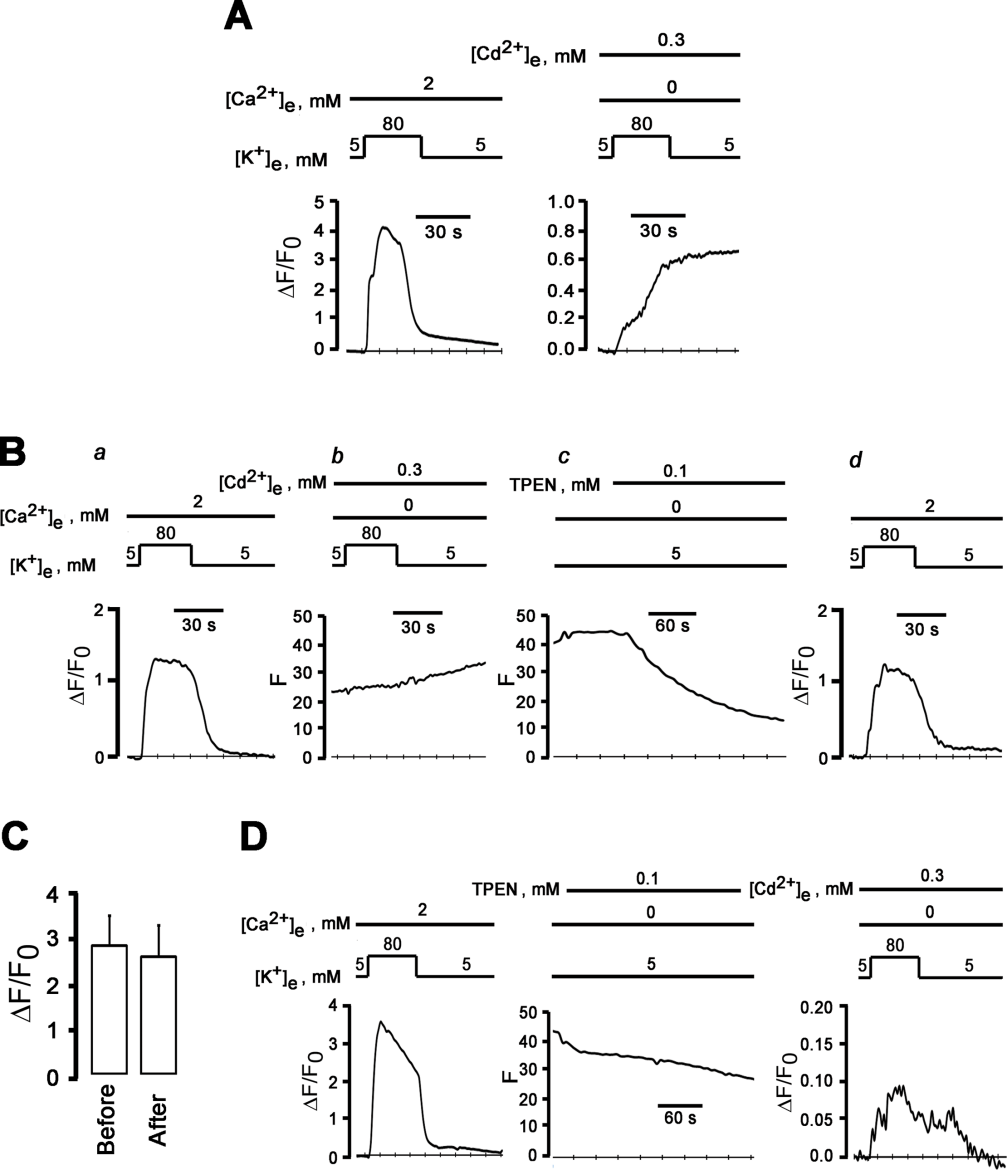
External Cd^2+^ does not suppress high [K^+^]_e_-induced ΔF/F_o_ transients in 0 mM [Ca^2+^]_e_. **A**: Irreversible increase of ΔF/F_0_ in depolarized neurons in the presence of extracellular Cd^2+^. Shown are time courses of changes in cytosolic ΔF/F_0_ elicited by 80 mM [K^+^]_e_ in the presence of 2 mM Ca^2+^ (left panel) and by 80 mM [K^+^]_e_ and 300 μM Cd^2+^ in a Ca^2+^-free bath solution (right panel). The increase of ΔF/F_0_ does not recover after removal of high-[K^+^]_e_-induced depolarization. **B:** External cadmium does not block depolarization-induced increase in cytosolic ΔF/F_0_ in the absence of extracellular Ca^2+^. (*a*) reversible increase in ΔF/F_0_ in response to a 30-s exposure to 80 mM [K^+^]_e_ in 2-mM Ca^2+^ bath solution. (*b*) and (*c*): a second exposure to 80 mM [K^+^]_e_ in Ca^2+^-free solution supplemented with 300 μM CdCl_2_ causes a sustained increase in fluo-4 fluorescence (*b*) which is not reversed until treatment of the cell with the membrane permeable metal chelator TPEN at 100 μM (*c*). A second K^+^ challenge of the TPEN-loaded neuron in a 2-mM Ca^2+^ bath solution (*d*) shows restoration of fluo-4 Ca^2+^ responsivity. **C**: Bar graphs summarizing mean ± SEM of peak cytosolic ΔF/F_0_ in sympathetic neurons before and after loading with TPEN (100 μM). *P* = 0.14 by paired *t*-test (n = 5 cells). **D**: Extracellular cadmium does not block depolarization-induced increase in ΔF/F_0_ in the absence of external Ca^2+^. Left panel: reversible increase in ΔF/F_0_ in response to a 30-s exposure to 80 mM [K^+^]_e_ in 2 mM [Ca^2+^]_e_. Following TPEN loading (middle panel), a second 30-s [K^+^]_e_ challenge in a Ca^2+^-free bath solution containing 300 μM Cd^2+^ gives rise to a small-amplitude ΔF/F_0_ transient (right panel).

Voltage-gated calcium channels become permeable to monovalent cations when extracellular calcium concentrations fall to sub-micromolar levels [23], suggesting the possibility that depolarization–induced [Ca^2+^]_i_ increases in the absence of external Ca^2+^ are triggered by influx of Na^+^ and/or K^+^ through open calcium channels under our experimental conditions. Accordingly, we next examined the effect of pharmacologically inhibiting ion flux through high voltage-gated Ca^2+^ channels on [K^+^]_e_-Ca^2+^ transients in 0 mM [Ca^2+^]_e_. Extracellular Cd^2+^ has been shown previously to potently block ion flux through neuronal, high voltage-gated calcium channels in a concentration-dependent manner [10], without altering channel gating. Our measurements of whole-cell Ca^2+^ currents in voltage-clamped neurons (using Ba^2+^ as the charge carrier) confirmed the absence of resolvable inward currents following the addition of 300 μM Cd^2+^ to the bath solution (S1A–S1C Fig), supporting the notion that Cd^2+^ at this concentration potently blocked currents through voltage-activated Ca^2+^ channels, consistent with previous studies in adult sympathetic ganglion neurons [9,24]. In addition, Cd^2+^ at concentrations that block ion flux has been reported to not affect membrane potential of sympathetic ganglion neurons [10]. Thus, extracellular Cd^2+^ in micromolar concentrations should be ideally suited to distinguish a role of ion flux through versus gating of voltage-dependent Ca^2+^ channels in mediating depolarization-induced rise in [Ca^2+^]_i_ in 0 mM [Ca^2+^]_e_. A typical ΔF/F_0_ response of a sympathetic ganglion neuron to a 30-s exposure to 80 mM [K^+^]_e_ and 300 μM Cd^2+^ in the absence of external Ca^2+^ is shown in Fig 7A. In these conditions, high [K^+^]_e_ still caused an increase in ΔF/F_0_, with no sign of recovery following return to 5 mM [K^+^]_e_. The irreversible increase in fluo-4 fluorescence resulted from Cd^2+^ influx into the cell, because it was completely reversed by a 5-min incubation with the membrane-permeant divalent metal chelator tetrakis (2-pyridylmethyl) ethylendiamine (TPEN; 100 μM; Fig 7B), whose affinity for Cd^2+^ (*K*_*d*_ = 10^−12^ M) [25] is several orders of magnitude higher than that for Ca^2+^ (*K*_*d*_ = 10^−4.4^ M) [26]. The means of high [K^+^]_e_-induced changes in peak ΔF/F_0_ in the presence of 2 mM [Ca^2+^]_e_ were not significantly different before and after loading with TPEN (Fig 7C), suggesting that TPEN can be used to selectively suppress the Cd^2+^ influx-dependent component of the fluo-4 signal in our experimental conditions. Indeed, combined exposure of another TPEN-loaded neuron to 80 mM [K^+^]_e_ and 300 μM Cd^2+^ in the absence of external Ca^2+^ revealed the typical ΔF/F_0_ response pattern seen in the absence of Cd^2+^, i.e., the increase in ΔF/F_0_ readily resolved following return to physiological [K^+^]_e_ (Fig 7D). Identical observations were made in 4 other TPEN-loaded neurons.

**Fig 8:**
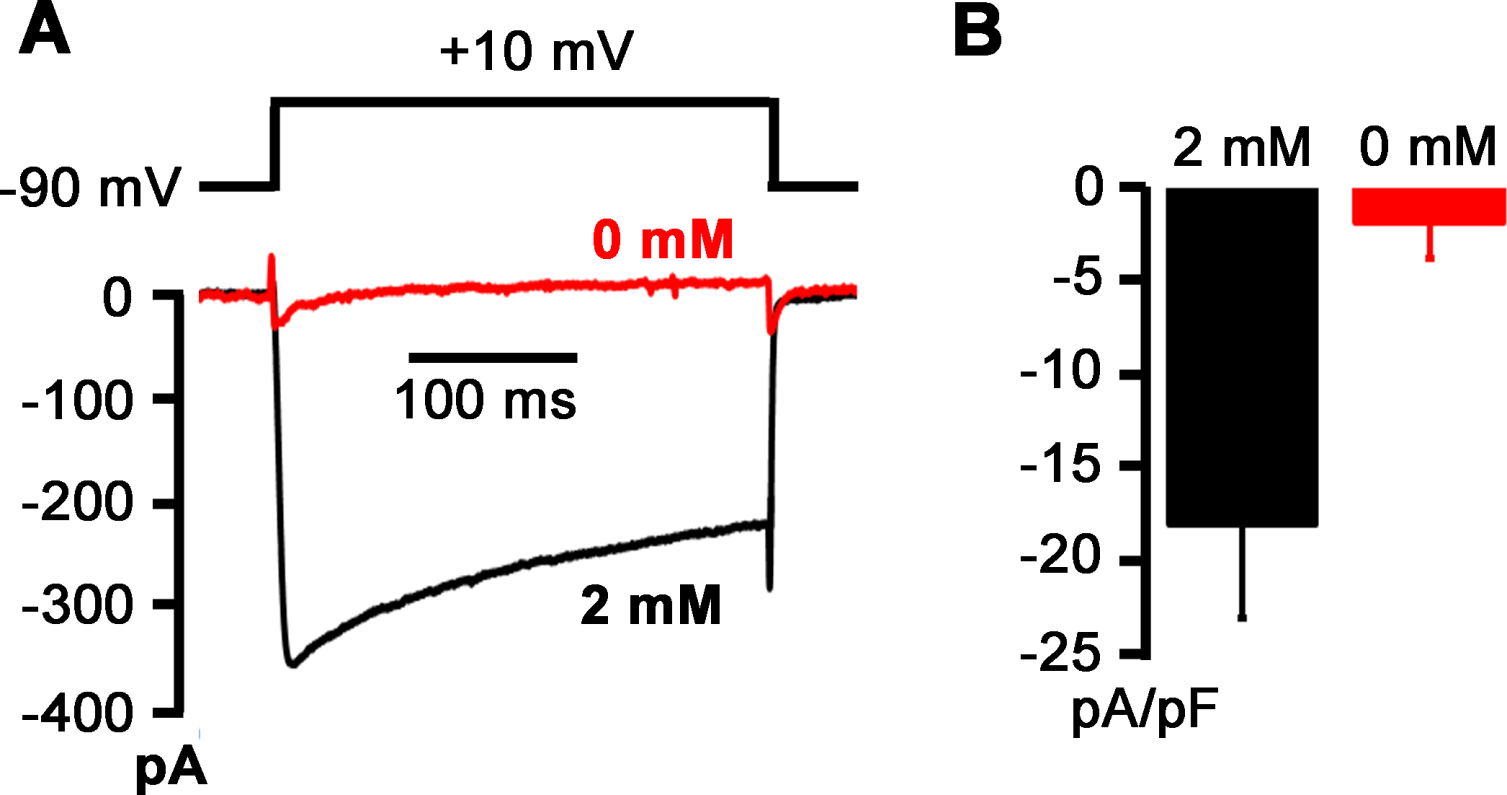
Voltage-gated Ca^2+^ channels do not conduct Na^+^ after removal of extracellular Ca^2+^. **A**: Exemplar whole-cell current traces sequentially recorded in a voltage-clamped neuron during 300-ms step depolarizations to +10 mV from a holding potential of -90 mV in 2 and 0 mM [Ca^2+^]_e_. The bath solution also contained 1 μM tetrodotoxin to block voltage-gated Na^+^ channels. Voltages were not corrected for liquid junction potential. Numbers in mM denote extracellular Ca^2+^ concentration. Voltage-clamp protocol is shown in the upper panel. **B**: Bar graph summarizing means of peak inward currents evoked by depolarizations to +10 mV in the presence and absence of external Ca^2+^ in the same cells. Error bars represent SEM (n = 4 cells). *P* = 0.02 versus 2 mM [Ca^2+^]_e_ by paired *t*-test.

To further examine whether voltage-dependent Ca^2+^ channels conduct Na^+^ currents in the absence of extracellular Ca^2+^, we sequentially measured macroscopic currents carried by these channels in 2 and 0 mM [Ca^2+^]_e_. Extracellular concentrations of Mg^2+^ (2 mM) and Na^+^ (140 mM) remained unchanged. Tetrodotoxin (1 μM) was present in the bath solution throughout the measurements to block voltage-gated Na^+^ channels. Exemplar whole-cell current traces acquired in response to step depolarizations to +10 mV before and after Ca^2+^ withdrawal are shown in Fig 8A. Removal of extracellular Ca^2+^ resulted in loss of inward currents, indicating that voltage-gated Ca^2+^ channels do not carry resolvable Na^+^ currents in the ionic conditions used here. Overall, these results support the notion that depolarization-induced rises in [Ca^2+^]_i_ in 0 mM [Ca^2+^]_e_ are not triggered by ion fluxes through high voltage-gated Ca^2+^ channels.

## Discussion

Our results indicate that the increase in global cytosolic [Ca^2+^] seen in response to prolonged depolarization in nominally Ca^2+^-free bath solution cannot be attributed to Ca^2+^ influx and subsequent Ca^2+^-induced Ca^2+^ release. Further, a skeletal muscle excitation-contraction–like mechanism as reported previously for hypothalamic magnocellular neurons, ischemically injured spinal cord white matter, and hippocampal neurons, can be excluded as the mechanism underlying depolarization-induced Ca^2+^ mobilization [3–5]. On the other hand, our findings are similar to those reported previously by Ryglewski et al. demonstrating that insect dorsal unpaired median neurons are capable of transducing incremental depolarization into gradual increases in cytosolic [Ca^2+^], involving Ca^2+^- influx-independent activation of a G protein-phospholipase C-IP_3_ receptor pathway. However, the nature of the voltage sensor was not identified in the latter study [6].

Na-induced dissociation of G-protein subunits has been demonstrated previously in neurons [27], suggesting the possibility that high [K^+^]_e_-evoked Ca^2+^ mobilization from IP_3_-sensitive stores as seen in the present study involves an ion influx-dependent, but not strictly depolarization-dependent, mechanism. Specifically, Na^+^ permeation of voltage-gated Ca^2+^ channels in the absence of external Ca^2+^ is one possible pathway linking Ca^2+^ release to membrane depolarization in our experimental conditions. We think this possibility to be unlikely for several reasons. First, extracellular Cd^2+^ at concentrations that completely blocked inward currents through voltage-gated Ca^2+^ channels, did not suppress high [K^+^]_e_-[Ca^2+^]_i_ transients in the absence of extracellular Ca^2+^. Second, we did not detect resolvable inward currents through voltage-dependent Ca^2+^ channels in voltage-clamped, depolarized neurons upon removal of extracellular Ca^2+^. Third, high [K^+^]_e_-Ca^2+^ transients in Ca^2+^-free bath solutions could be readily evoked in the presence of 1 mM extracellular Mg^2+^, which corresponds to 4 times the IC_50_ of Mg^2+^-induced block of Na^+^ current through N-type Ca^2+^ channels [28], the major subtype of voltage-gated Ca^2+^ channels in adult sympathetic ganglion neurons.

Also, ions other than Ca^2+^ can enter the cell via alternative voltage-modulated pathways, e.g., voltage-gated Na^+^ channels, or voltage-independent pathways, e.g., acetylcholine receptor and/or transient receptor potential cation channels. The gradual decrease in Na^+^ concentration in the bath solution for increasingly large depolarizations results in an incremental reduction in the Na^+^ driving force, progressively reducing its flux through voltage-independent channels as the membrane becomes more depolarized. This behavior conflicts with the non-monotonic dependence of depolarization-evoked peak ΔF/F_0_ amplitude on voltage that we observed in the absence of external Ca^2+^ (see Fig 2E). It thus appears unlikely that the magnitude of Na^+^ movement through voltage-independent pathways constitutes the trigger for Ca^2+^ mobilization in our experiments. Because the relationship between changes in membrane potential and those in extracellular K^+^ concentration followed the Nernst equation with a slope close to that expected for a K^+^-selective ion channel (see Fig 2C), the K^+^ net flux at each membrane potential achieved with various [K^+^]_e_ must have been zero or close to zero, excluding voltage-dependent changes in transmembrane K^+^ flux as a possible regulator of Ca^2+^ release in 0 mM [Ca^2+^]_e_.

Finally_,_ the possibility that the voltage-dependence of peak ΔF/F_0_ magnitude in the absence of external Ca^2+^ reflects the voltage-dependence of a steady-state current through voltage-gated Na^+^ channels has to be considered. Previous studies by others revealed the existence of a non-inactivating component of the Na^+^ current in neurons [29], whose magnitude exhibits a non-monotonic, U-shaped, dependence on voltage. However, these persistent, so-called ‘window’ Na^+^ currents activated at voltages significantly more negative than those of peak ΔF/F_0_ in 0 mM [Ca^2+^]_e_, making persistent Na^+^ currents an unlikely candidate linking Ca^2+^ discharge to membrane voltage changes. Overall, our results thus support the notion that depolarization *per se* suffices to trigger Ca^2+^ discharge from IP_3_-sensitive stores in 0 mM [Ca^2+^]_e._

The inverse U-shaped dependence of peak ΔF/F_0_ amplitude on membrane potential in 0 mM [Ca^2+^]_e_ is both unexpected and puzzling. Because peak *P*_*act*_ of voltage-gated Ca^2+^ channels steeply increases with depolarization over the range of membrane potentials we studied, we can exclude the possibility that gating changes as are typically achieved during peak activation of these channels, underlie voltage-induced, Ca^2+^ influx-independent Ca^2+^ release in our experiments. However, the observed non-monotonic behavior of Ca^2+^ signal strength with increasing depolarization is compatible with a mechanism, wherein the voltage-dependence of a channel’s steady-state, but not peak, *P*_*act*_ serves to transduce prolonged changes in membrane potential into graded Ca^2+^ mobilization. Indeed, our numerical simulations using experimentally determined activation and inactivation properties of voltage-activated Ca^2+^ channels demonstrate that the channel’s voltage-dependence of steady-state *P*_*act*_ on membrane potential overlaps with that of peak ΔF/F_0_ magnitude measured in 0 mM [Ca^2+^]_e_. Direct proof of the role of conformational changes of a Ca^2+^ channel complex in tuning Ca^2+^ discharge from IP_3_-sensitive internal stores will require experimental immobilization of gating charges in subtypes of voltage-gated Ca^2+^ channels known to be expressed in adult sympathetic ganglion neurons, including N- and P/Q-type Ca^2+^ channels. It will also require measurements of absolute free [Ca^2+^]_i_ levels. Because we have not calibrated the fluo-4 signal for our experimental conditions, we were unable to directly correlate changes in membrane potential to those in free [Ca^2+^]_i_.

Besides voltage-gated ion channels, it is possible that non-ion channel proteins act as voltage sensors, e.g., the Na-K ATPase, the Na-Ca exchanger or the recently discovered voltage-sensitive phosphatide phosphatase, although expression of the latter in mammalian neuronal tissue has not yet been confirmed [30]. Additional studies are needed to identify the molecule capable of transducing the electrical signal into Ca^2+^ discharge from IP_3_-sensitive stores in adult sympathetic neurons.

It was demonstrated previously that insect dorsal unpaired median neurons possess a membrane voltage sensor that, independent of Ca^2+^ influx, causes G-protein activation, which subsequently leads to Ca^2+^ release from intracellular stores via phospholipase C and IP_3_-receptor activation [6]. It remains to be determined whether the voltage-sensitive Ca^2+^ release mechanism in our study utilizes the same signaling pathway or whether the plasmalemmal voltage-sensor directly interacts with the IP_3_ receptor in the ER membrane. The slow rise in cytosolic [Ca^2+^] during prolonged depolarizations in the absence of external Ca^2+^ suggests the involvement of intermediary steps in transducing the electrical signal into a Ca^2+^ release from intercellular stores. Moreover, dihydropyridine receptors have previously been reported to act as voltage sensors for a voltage-dependent, IP_3_ receptor-mediated, slow Ca^2+^ signal in skeletal muscle cells [20]. A model was proposed in which the dihydropyridine receptor decodes the electrical signal into G-protein-dependent activation of phospholipase C to produce IP_3_, which then diffuses to IP_3_ receptors located on the ER and nuclear membrane, ultimately activating intracellular signaling cascades. Although our experiments provide no evidence for a role of dihydropyridine receptors as voltage-sensors in depolarization-evoked Ca^2+^ release in sympathetic ganglion neurons, our data suggests that both cell types share the signaling events downstream of their respective voltage-sensor.

Although 2-APB has been shown previously to inhibit IP_3_-mediated Ca^2+^ release in neurons [31], it also exerts unspecific effects on Ca^2+^ entry in non-excitable cell types, e.g. via blockade of cation-selective channels encoded by the by transient receptor potential (TRP) genes [32]. However, our observation that xestospongin C at a concentration that has been shown previously to specifically inhibit IP_3_ receptor signaling in a variety of mammalian cell types [33,34], similarly suppressed depolarization-induced increases in [Ca^2+^]_i_ in the absence of external Ca^2+^ support our conclusion that depolarization–evoked rises in [Ca^2+^] require functional IP_3_ receptors.

### Potential function of voltage-induced Ca^2+^ release in sympathetic neurons

Voltage-induced Ca^2+^ release constitutes a novel mechanism by which adult sympathetic ganglion neurons couple electrical activity to graded rises in intracellular [Ca^2+^]. The extent to which this mechanism contributes to the increase in [Ca^2+^] that normally occurs in response to single or repetitive action potentials remains to be quantitated. Eltit and co-workers previously demonstrated that tetanic stimulation of skeletal myotubes in the absence of extracellular Ca^2+^ gives rise to long-lasting, IP_3_-generated, slow Ca^2+^ signals both in the nucleus and cytoplasm [34]. It will be interesting to determine whether repetitive electrical discharge of sympathetic ganglion neurons, such as occurring physiologically *in situ*, also results in slow Ca^2+^ signals similar to those evoked by high [K^+^]_e_ in the present study.

Although the magnitude of the depolarization-induced global Ca^2+^ transient is small compared to that of a transient elicited in normal [Ca^2+^]_e_, it is possible that Ca^2+^ is released into microdomains in which it may exert strong effects on exocytosis and/or Ca^2+^-sensitive ion channels and enzymes, ultimately altering excitability, energy homeostasis, and transcriptional activity of the neuron. With regard to the latter, the elevations in nuclear fluo-4 fluorescence that were observed to occur concomitantly with those in the cytosol both in 2 and 0 mM [Ca^2+^]_i_, may play a role in transcriptional regulation [10]. The magnitude of depolarization-induced changes in nuclear fluo-4 fluorescence intensity in 2 mM [Ca^2+^]_e_ often reached saturation using gain settings that were optimized to monitor cytosolic fluorescence, precluding simultaneous measurements in both compartments.

Small elevations in cytosolic Ca^2+^ like those arising as a consequence depolarization-evoked IP_3_-recpetor stimulation, may enhance the Ca^2+^ -sensitivity of nearby ryanodine receptors, thereby converting the cytoplasm in an excitable medium capable of producing regenerative Ca^2+^ responses.

## Supporting Information

S1 Fig Effect of cadmium, a non-selective blocker of voltage-gated Ca^2+^ channels, on high [K^+^]_e_-induced cytosolic Ca^2+^ transients in postganglionic sympathetic neurons. **A** and **B**: Family of current traces recorded from an isolated sympathetic neuron in the absence (A) and presence of 300 μM CdCl_2_ in the external solution. Currents were evoked by 200-ms voltage steps, ranging from -70 to +50 mV in 10-mV increments. **C**: Peak *IBa–*voltage relationship for the cadmium-sensitive (circles) and–resistant (squares) currents shown in A and B. CdCl2 eliminated all inward currents.
(TIF)

## References

[1] Berridge MJ. Neuronal calcium signaling. Neuron. 1998; 21(1): 13–26. 9697848 10.1016/s0896-6273(00)80510-3

[2] Grienberger C, Konnerth A. Imaging calcium in neurons. Neuron. 2012; 73(5): 862–885. 10.1016/j.neuron.2012.02.011 22405199

[3] Kim S, Yun HM, Baik JH, Chung KC, Nah SY, Rhim H. Functional interaction of neuronal Cav1.3 L-type calcium channel with ryanodine receptor type 2 in the rat hippocampus. J Biol Chem. 2007; 282(45): 32877–32889. 17823125 10.1074/jbc.M701418200

[4] De Crescenzo V, Fogarty KE, Zhuge R, Tuft RA, Lifshitz LM, Carmichael J, et al Dihydropyridine receptors and type 1 ryanodine receptors constitute the molecular machinery for voltage-induced Ca^2+^ release in nerve terminals. J Neurosci. 2006; 26(29): 7565–7574. 16855084 10.1523/JNEUROSCI.1512-06.2006 PMC6674279

[5] Ouardouz M, Nikolaeva MA, Coderre E, Zamponi GW, McRory JE, Trapp BD, et al Depolarization-induced Ca^2+^ release in ischemic spinal cord white matter involves L-type Ca^2+^ channel activation of ryanodine receptors. Neuron. 2003; 40(1): 53–63. 14527433 10.1016/j.neuron.2003.08.016

[6] Ryglewski S, Pflueger HJ, Duch C. Expanding the neuron's calcium signaling repertoire: intracellular calcium release via voltage-induced PLC and IP_3_R activation. PLoS Biol. 2007; 5(4): e66 17341135 10.1371/journal.pbio.0050066 PMC1808487

[7] Kukwa W, Macioch T, Szulczyk PJ. Stellate neurones innervating the rat heart express N, L and P/Q calcium channels. J Auton Nerv Syst. 1998; 74(2–3): 143–151. 9915630 10.1016/s0165-1838(98)00154-4

[8] Namkung Y, Smith SM, Lee SB, Skrypnyk NV, Kim HL, Chin H, et al Targeted disruption of the Ca^2+^ channel beta3 subunit reduces N- and L-type Ca^2+^ channel activity and alters the voltage-dependent activation of P/Q-type Ca^2+^ channels in neurons. Proc Natl Acad Sci U S A. 1998; 95(20): 12010–12015. 9751781 10.1073/pnas.95.20.12010 PMC21756

[9] Martínez-Pinna J, Lamas JA, Gallego R. Calcium current components in intact and dissociated adult mouse sympathetic neurons. Brain Res. 2002; 951(2): 227–236. 12270501 10.1016/s0006-8993(02)03165-7

[10] Wheeler DG, Barrett CF, Groth RD, Safa P, Tsien RW. CaMKII locally encodes L-type channel activity to signal to nuclear CREB in excitation-transcription coupling. J Cell Biol. 2008; 183(5): 849–863. 10.1083/jcb.200805048 19047462 PMC2592819

[11] Akita T, Kuba K. Functional triads consisting of ryanodine receptors, Ca^2+^ channels, and Ca^2+^-activated K^+^ channels in bullfrog sympathetic neurons. Plastic modulation of action potential. J Gen Physiol. 2000; 116(5): 697–720. 11055998 10.1085/jgp.116.5.697 PMC2229477

[12] Albrecht MA, Colegrove SL, Hongpaisan J, Pivovarova NB, Andrews SB, Friel DD. Multiple modes of calcium-induced calcium release in sympathetic neurons I: attenuation of endoplasmic reticulum Ca^2+^ accumulation at low [Ca^2+^]_i_ during weak depolarization. J Gen Physiol. 2001; 118(1): 83–100. 11429446 10.1085/jgp.118.1.83 PMC2233742

[13] Li BY, Schild JH. Electrophysiological and pharmacological validation of vagal afferent fiber type of neurons enzymatically isolated from rat nodose ganglia. J Neurosci Methods. 2007; 164(1): 75–85. 17512602 10.1016/j.jneumeth.2007.04.003 PMC2003207

[14] Friel DD, Tsien RW. A caffeine- and ryanodine-sensitive Ca^2+^ store in bullfrog sympathetic neurones modulates effects of Ca^2+^ entry on [Ca^2+^]_i_. J Physiol. 1992; 450: 217–246. 1432708 10.1113/jphysiol.1992.sp019125 PMC1176120

[15] Schoenmakers TJ, Visser GJ, Flik G, Theuvenet AP. CHELATOR: an improved method for computing metal ion concentrations in physiological solutions. Biotechniques. 1992; 12(6): 870–879. 1642895

[16] Estève E, Eltit JM, Bannister RA, Liu K, Pessah IN, Beam KG, et al A malignant hyperthermia-inducing mutation in RYR1 (R163C): alterations in Ca^2+^ entry, release, and retrograde signaling to the DHPR. J Gen Physiol. 2010; 135(6): 619–628. 10.1085/jgp.200910328 20479110 PMC2888056

[17] Satoh H, Delbridge LM, Blatter LA, Bers DM. Surface: volume relationship in cardiac myocytes studied with confocal microscopy and membrane capacitance measurements: species-dependence and developmental effects. Biophys J. 1996; 70(3): 1494–1504. 8785306 10.1016/S0006-3495(96)79711-4 PMC1225076

[18] Thayer SA, Hirning LD, Miller RJ. The role of caffeine-sensitive calcium stores in the regulation of the intracellular free calcium concentration in rat sympathetic neurons in vitro. Mol Pharmacol. 1988; 34(5): 664–673. 3193957

[19] Beam KG, Bannister RA. Looking for answers to EC coupling's persistent questions. J Gen Physiol. 2010; 136(1): 7–12. 10.1085/jgp.201010461 20584887 PMC2894545

[20] Araya R, Liberona JL, Cárdenas JC, Riveros N, Estrada M, Powell JA, et al Dihydropyridine receptors as voltage sensors for a depolarization-evoked, IP_3_R-mediated, slow calcium signal in skeletal muscle cells. J Gen Physiol. 2003; 121(1): 3–16. 12508050 10.1085/jgp.20028671 PMC2217318

[21] Nimmrich V, Gross G. P/Q-type calcium channel modulators. Br J Pharmacol. 2012; 167(4): 741–759. 10.1111/j.1476-5381.2012.02069.x 22670568 PMC3575775

[22] Bannister RA, Pessah IN, Beam KG. The skeletal L-type Ca^2+^ current is a major contributor to excitation-coupled Ca^2+^ entry. J Gen Physiol. 2009; 133(1): 79–91. 10.1085/jgp.200810105 19114636 PMC2606935

[23] Hille B. Ion channels of excitable membranes. 3rd ed. Sunderland: Sinauer Associates, MA; 2001.

[24] Belluzzi O, Sacchi O. Calcium currents in the normal adult rat sympathetic neurone. J Physiol. 1989; 412: 493–512. 2557430 10.1113/jphysiol.1989.sp017628 PMC1190588

[25] Usai C, Barberis A, Moccagatta L, Marchetti C. Pathways of cadmium influx in mammalian neurons. J Neurochem. 1999; 72(5): 2154–2161. 10217297 10.1046/j.1471-4159.1999.0722154.x

[26] Hinkle PM, Shanshala ED 2nd, Nelson EJ. Measurement of intracellular cadmium with fluorescent dyes. Further evidence for the role of calcium channels in cadmium uptake. J Biol Chem. 1992; 267(35): 25553–25559. 1281160

[27] Blumenstein Y, Maximyuk OP, Lozovaya N, Yatsenko NM, Kanevsky N, Krishtal O, et al Intracellular Na^+^ inhibits voltage-dependent N-type Ca^2+^ channels by a G protein betagamma subunit-dependent mechanism. J Physiol. 2004; 556(Pt 1): 121–134. 14742725 10.1113/jphysiol.2003.056168 PMC1664899

[28] Polo-Parada L, Korn SJ. Block of N-type calcium channels in chick sensory neurons by external sodium. J Gen Physiol. 1997; 109(6): 693–702. 9222896 10.1085/jgp.109.6.693 PMC2217043

[29] Parri HR, Crunelli V. Sodium current in rat and cat thalamocortical neurons: role of a non-inactivating component in tonic and burst firing. J Neurosci. 1998; 18(3): 854–867. 9437007 10.1523/JNEUROSCI.18-03-00854.1998 PMC6792749

[30] Okamura Y, Murata Y, Iwasaki H. Voltage-sensing phosphatase: actions and potentials. J Physiol. 2009; 587(Pt 3): 513–520. 10.1113/jphysiol.2008.163097 19074969 PMC2670076

[31] Bootman MD, Collins TJ, Mackenzie L, Roderick HL, Berridge MJ, Peppiatt CM. 2-aminoethoxydiphenyl borate (2-APB) is a reliable blocker of store-operated Ca^2+^ entry but an inconsistent inhibitor of InsP_3_-induced Ca^2+^ release. FASEB J. 2002; 16(10): 1145–1150. 12153982 10.1096/fj.02-0037rev

[32] Xu SZ, Zeng F, Boulay G, Grimm C, Harteneck C, Beech DJ. Block of TRPC5 channels by 2-aminoethoxydiphenyl borate: a differential, extracellular and voltage-dependent effect. Br J Pharmacol. 2005; 145(4): 405–414. 15806115 10.1038/sj.bjp.0706197 PMC1576154

[33] Bofill-Cardona E, Vartian N, Nanoff C, Freissmuth M, Boehm S. Two different signaling mechanisms involved in the excitation of rat sympathetic neurons by uridine nucleotides. Mol Pharmacol. 2000; 57(6): 1165–1172. 10825387

[34] Eltit JM, Hidalgo J, Liberona JL, Jaimovich E. Slow calcium signals after tetanic electrical stimulation in skeletal myotubes. Biophys J. 2004; 86(5): 3042–3051. 15111418 10.1016/S0006-3495(04)74353-2 PMC1304170

