# Supplementary figures and images for: Voltage-Induced Ca2+ Release in Postganglionic Sympathetic Neurons in Adult Mice
Source: PLoS One. 2016 Feb 9;11(2):e0148962. doi: 10.1371/journal.pone.0148962 (PMC4747524; doi:10.1371/journal.pone.0148962)

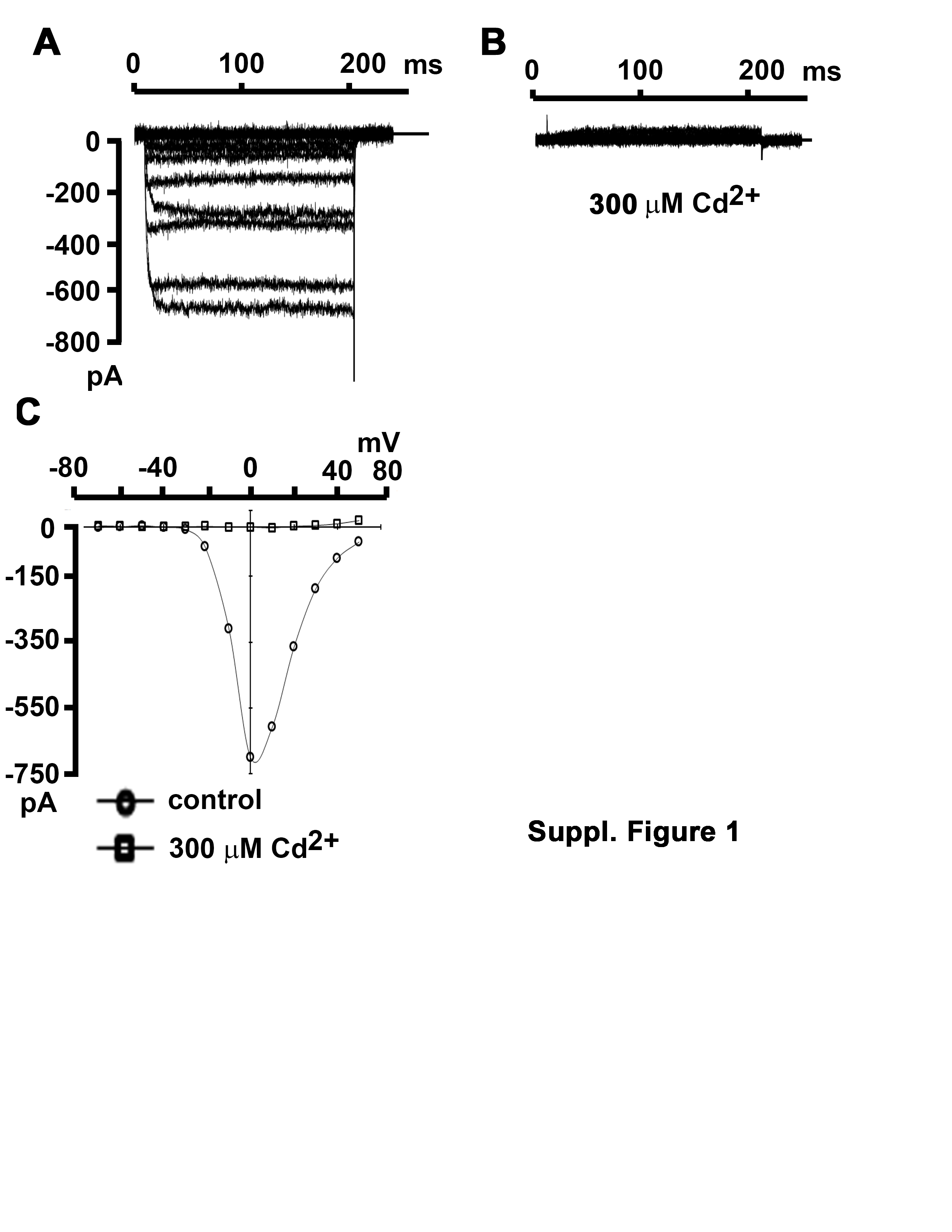

Supplement: S1 Fig — A and B: Family of current traces recorded from an isolated sympathetic neuron in the absence (A) and presence of 300 μM CdCl2 in the external solution. Currents were evoked by 200-ms voltage steps, ranging from -70 to +50 mV in 10-mV increments. C: Peak IBa–voltage relationship for the cadmium-sensitive (circles) and–resistant (squares) currents shown in A and B. CdCl2 eliminated all inward currents. (TIF) [file pone.0148962.s001.tif]
